# Individually addressable nanoscale OLEDs

**DOI:** 10.1126/sciadv.adz8579

**Published:** 2025-10-22

**Authors:** Cheng Zhang, Björn Ewald, Leo Siebigs, Luca Steinbrecher, Maximilian Rödel, Thomas Fleischmann, Monika Emmerling, Jens Pflaum, Bert Hecht

**Affiliations:** ^1^ Experimental Physics 5, University of Würzburg, Am Hubland, 97074 Würzburg, Germany.; ^2^ Experimental Physics 6, University of Würzburg, Am Hubland, 97074 Würzburg, Germany.

## Abstract

When reducing pixel size below the wavelength of light, the conventional stacked geometry of organic light-emitting diodes (OLEDs) is dominated by sharp nanoelectrode contours. This causes spatially imbalanced charge carrier transport and recombination resulting in low external quantum efficiency (EQE) and filament formation accelerating device failure. Here, we circumvent these limitations by selectively passivating nanoelectrode edges with an insulating layer, while simultaneously defining a nanoaperture in flat areas. We thereby ensure controlled charge carrier recombination and suppress filament growth. After demonstrating efficient hole injection by gold nanoelectrodes, we realize individually addressable subwavelength OLED pixels (300 nanometers by 300 nanometers) based on plasmonic gold patch antennas for light extraction. We achieve an EQE of 1%, a maximum luminance of 3000 candela per square meter, and fast response times exceeding video rates. Our results highlight a scalable strategy to overcome key electronic and optical bottlenecks of nanoscale optoelectronic devices and demonstrate the potential of plasmonic patch antennas for high-density, high-performance OLED integration.

## Teaser

Individually addressable subwavelength OLED pixels use plasmonic patch antenna electrodes for light extraction.

## INTRODUCTION

In organic semiconductor technologies, vertical multilayer architectures offer precise control over optoelectronic properties, with applications ranging from organic light-emitting diodes (OLEDs) (*1*–*3*) to organic photodetectors (*4*) and vertical organic transistors (*5*). Recent technological advancements are primarily driven by an ongoing miniaturization of these components. Examples include miniaturized transistor structures for lab-on-a-chip systems (*6*–*8*), micro-OLEDs for optogenetic stimulation (*9*), and most prominent, micro-OLED pixels for virtual reality (VR) and augmented reality (AR) displays (*10*–*14*). Being used in wearables, AR and VR displays in general require small form factors and low power consumption without compromising the viewing experience by the so-called screen-door effect. This requires pixel densities of more than 6000 pixels per inch (*15*, *16*). While inorganic LEDs offer high brightness and are well suited for AR applications, they face substantial manufacturing challenges and efficiency losses when scaled down to the (sub)micrometer regime (*17*–*23*). In contrast, OLEDs have become a mainstream solution due to their scalable fabrication, cost-effectiveness, and compatibility with semiconductor processes (*15*). Moreover, their Lambertian emission characteristics and the high exciton binding energies (up to 1 eV) in organic semiconductors simplify downscaling (*24*). Micro-OLED technology is based on top-emitting OLED pixels fabricated on complementary metal-oxide semiconductor (CMOS) backplanes, with the smallest reported pixel sizes between 1 and 10 μm (*10*–*14*).

**Fig. 1.**
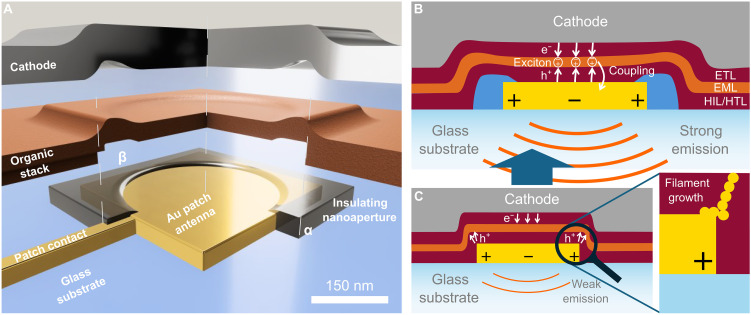
Conceptual design of nano-OLED pixels with an individually addressable bottom nanoelectrode. (**A**) Sketch of the device architecture (cut open explosion view). A subwavelength Au patch electrode supporting plasmonic modes is used as bottom anode. To bypass the detrimental effects of electrode downscaling the edges and corners of the nanoelectrode are insulated, leaving a centered nanoaperture with an area of homogenous electric field distribution (see “insulating nanoaperture”). A standard organic stack consisting of hole injection layer (HIL), hole transport layer (HTL), emissive layer (EML), and electron transport layer (ETL) is applied on top, followed by an extended metallic top cathode. (**B**) Cut through (A) along α-β. Spatially controlled hole injection (bottom anode) and electron injection (top cathode) lead to exciton formation above the nanoaperture within the emissive layer. Upon recombination, excitons couple to the plasmonic mode(s) of the Au nanopatch, leading to strong emission. (**C**) Device without nanoaperture: Inhomogeneous charge carrier injection leads to poor emission and promotes filament growth (see inset to the right).

Here, we strive for subwavelength pixels for applications in sophisticated near-eye displays (e.g., light-field displays) (*25*) and in photonic integrated circuits. Subwavelength miniaturization entails critical scaling effects, making it virtually impossible to simply downscale devices without accounting for these phenomena (*26*). In organic devices, only a few studies have addressed charge carrier transport and recombination in nanoscale junctions, but none have implemented nanoelectrodes, which are essential for the individual driving of nanoscale OLEDs in display applications (*27*–*31*). However, individual addressing poses key challenges. Sharp edges at nanoelectrodes create intense local electric fields that distort Schottky barriers and, thereby, modify or even change charge carrier injection mechanisms. For example, the dominant tunneling contribution to the overall charge carrier injection for diodes of <100 nm imposes severe limitations on the performance of devices based on organic semiconductors with low charge carrier mobilities (*32*–*34*). Local electric field enhancement can lead to current density hotspots and imbalanced charge transport throughout the device (see Fig. 1C) (*35*) and to metallic filament formation, causing unstable operation if not failure of the entire device (see Fig. 1C, inset) (*36*–*38*). These effects can be further amplified by lithographic edge defects common in nanofabricated electrodes (*39*). Miniaturization also limits light output. The emitted power of an OLED pixel decreases with (*l*/λ)^2^, where *l* is the pixel size and λ is the emission wavelength, leading to severe optical inefficiencies at the subwavelength scale (*40*). We recently reported the integration of active subwavelength plasmonic nanoantenna electrodes into laterally arranged OLED nanopixels (nano-OLEDs) and demonstrated enhanced emission by means of coupling effects to the antennas (*41*). However, devices suffered from local field–induced filament growth and subsequent premature device failure and state-of-the-art multilayer organic stack designs with all their benefits could not be used (*41*, *42*).

Here, we present an OLED architecture that combines vertical device stacking with laterally defined plasmonic nanoantenna electrodes while suppressing effects of field hotspots at sharp edges. In contrast to earlier works that use passive plasmonic scatterers integrated into standard OLEDs only to counteract waveguiding (*43*–*45*), our concept, illustrated in Fig. 1 (A and B), thus enables individually addressable, stable, and efficient subwavelength pixels.

We use an insulating layer covering the electrode but featuring a nanoaperture in its center to suppress hole injection from nanoelectrode edges by insulating its peripheral corners and edges (Fig. 1, A and B). This effectively mitigates detrimental effects caused by electric field hotspots in these regions (Fig. 1C) and results in stable device operation with balanced charge carrier transport and recombination dynamics (Fig. 1B). Excitons on the emitter sites couple to the plasmonic mode(s) of the Au patch antenna used as the bottom anode, which radiates through the glass substrate into the far field. We validate this approach by first demonstrating nanoaperture-controlled hole injection through single Au nanoelectrodes in hole-only devices, followed by the demonstration of subwavelength individually addressable vertically stacked nano-OLEDs (300 nm by 300 nm). These nanopixels are stable even at high current densities and achieve external quantum efficiencies (EQEs) in the 1% range, a maximum luminance of 3000 cd m^−2^, and pixel switching speeds surpassing standard video frame rates (e.g., 60 frames s^−1^). Our methodology represents a notable advance in the miniaturization of optoelectronic devices by demonstrating precise spatial control of the charge carrier paths and, thus, of the active recombination and emissive regions in nanoscale OLED architectures and by leveraging key plasmonic nanoantenna effects in Au nanoelectrodes. Hence, it holds substantial implications, e.g., for the development of next-generation ultrasmall but high-resolution displays and other nano-optoelectronic systems (*46*–*48*).

## RESULTS

We first demonstrate hole-only devices based on 1 μm–by–1 μm Au patches to establish the use of gold as efficient anode material. To implement complete OLEDs, we then downscale individually addressable Au patches to 300 nm by 300 nm to allow for light outcoupling via the nanoelectrode’s plasmonic patch antenna modes.

### Au nanoelectrodes with nanoaperture

**Fig. 2.**
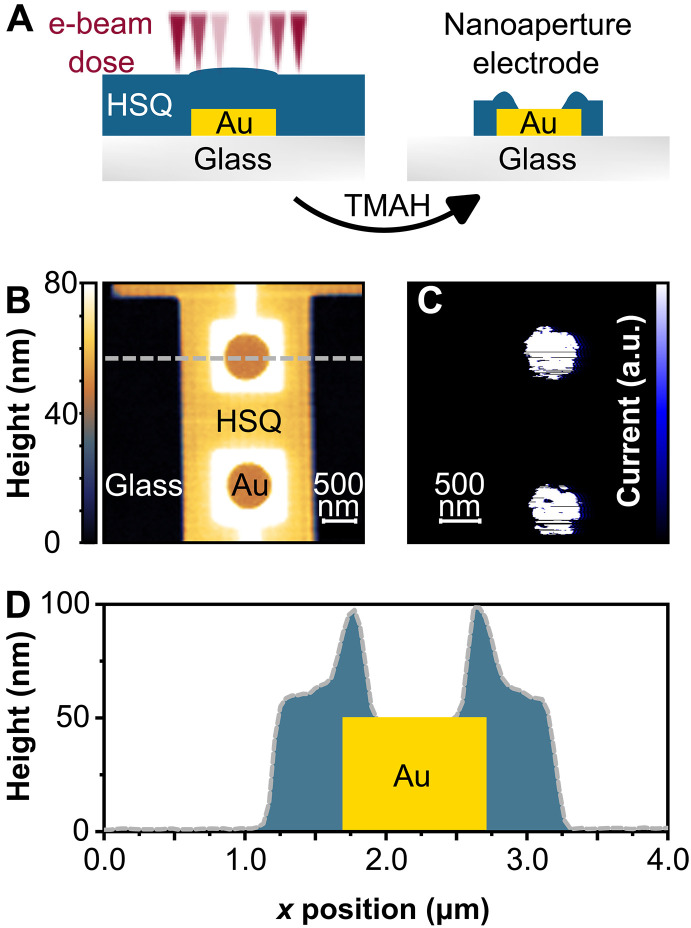
Au nanoelectrodes with nanoapertures. (**A**) EBL process with HSQ (blue) as negative resist covering the electrode. Cross-linking is controlled by a gradient e-beam dose indicated by the fading red color. Alkalic development with tetramethylammonium hydroxide (TMAH) results in the removal of unexposed HSQ and partial etching of exposed HSQ according to the degree of cross-linking. (**B**) Tapping-mode AFM images of two adjacent 1 μm–by–1 μm Au electrodes with individual electrical connectors. The electrode edges and the connector are fully covered by HSQ apart from a central 550-nm-diameter nanoaperture. (**C**) Conductive AFM image of the same area as in (B), revealing current injection only inside the apertures. a.u., arbitrary units. (**D**) Height profile along the dashed gray line in (B).

Electrostatic simulations of a 1 μm–by–1 μm quadratic electrode patch (fig. S1) suggest a threefold electric field enhancement at the electrode edges and a sixfold electric field enhancement at the corners compared to the electrode center. Quadratic Au nanoelectrodes (1 μm by 1 μm) are fabricated using standard electron beam lithography (EBL) and thermal metal evaporation. To achieve a uniform electric field that enables controlled charge injection in flat areas of the electrode, a high-quality thin metal film with minimal surface roughness is crucial. We use an evaporation rate of 1.5 nm s^−1^ in a high vacuum environment to deposit a 50-nm Au film resulting in a root mean square surface roughness of 1 nm (see fig. S2). To suppress particle attachment and the formation of rough metallic edges inherent to conventional EBL processes, we make use of a poly(methyl methacrylate) (PMMA) bilayer (PMMA 600K/950K) as positive resist. This approach generates a controlled undercut profile during resist development, thus effectively minimizing structural defects at the nanoelectrode edges after liftoff [see atomic force microscopy (AFM) and scanning electron microscopy (SEM) images in fig. S3]. To insulate edges and corners, we use a second EBL process using the high-resolution negative resist hydrogen silsesquioxane (HSQ) (*49*) to create an insulating layer with a centered nanoaperture on top of the Au nanoelectrode, as shown in Fig. 2.

To this end, we apply a gradient electron beam dose across the entire antenna pixel, ranging from zero at the electrode center to full dosage at the electrode edges (Fig. 2A). This results in a precise control of the shape and depth of the resulting central nanoaperture after the subsequent removal of unexposed HSQ resist, using tetramethylammonium hydroxide (TMAH). A successful opening of a nanoaperture is confirmed by tapping mode AFM, as displayed in Fig. 2 (B and D). In addition, the remaining electrical connection pad is insulated to prevent any potential leakage currents. To achieve a clean, open nanoaperture, we apply a mild oxidative etching step to the exposed Au portion of the electrode, using a highly diluted Lugol’s solution. Furthermore, conductive AFM measurements are carried out, as illustrated in Fig. 2C, which shows that the conductive area coincides precisely with the nanoaperture and, thus, allows for laterally defined hole injection. Our approach is equally effective for smaller electrode patches of 300 nm by 300 nm (see fig. S4).

### Hole-only devices

**Fig. 3.**
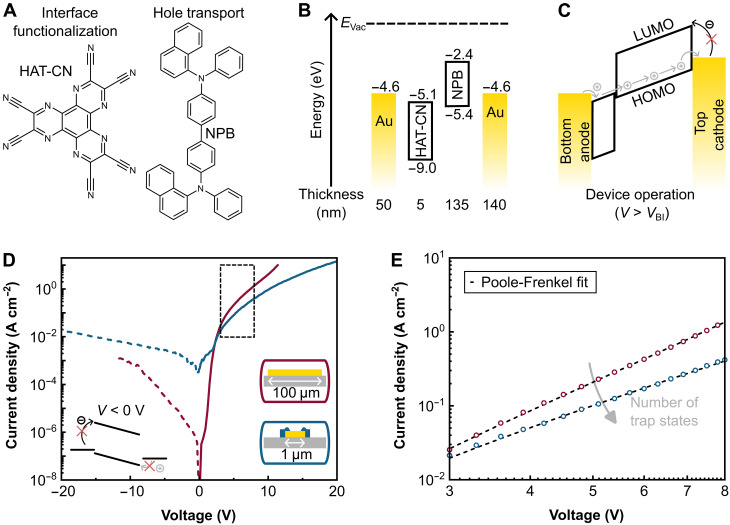
Electrical properties and device configuration of hole-only macrojunctions (electrode patch, 100 μm by 100 μm; active area, 1.0 × 10^−4^ cm^2^) and nanojunctions (electrode patch, 1 μm by 1 μm; nanoaperture diameter, 550 nm; active area, 2.4 × 10^−9^ cm^2^). (**A**) Molecular structures of the interface functionalization material HAT-CN and the hole transport material NPB. (**B**) Device architecture consisting of an Au bottom anode (50 nm in thickness) functionalized with an ultrathin HAT-CN layer (5 nm), followed by a hole transport layer consisting of NPB (135 nm) and the electron-blocking Au cathode (140 nm). The flat-band energy landscape does not consider Fermi level pinning of HAT-CN and NPB. (**C**) Principle of operation under an applied voltage *V* exceeding the built-in voltage *V*_BI_. Hole injection is mediated by the bottom electrode in conjunction with the HAT-CN interface layer, and injected holes are transported via the HOMO of NPB. Electron injection from the top cathode is prevented through the large energy barrier. (**D**) Semilogarithmic current density-voltage characteristics of representative macrojunction (red lines and inset to the right) and nanojunction (blue lines and inset to the right) diodes. The blocking character of both electrodes under reverse bias is highlighted by the inset to the left. (**E**) Corresponding double logarithmic presentation of the space charge–limited current and Poole-Frenkel fits in the regime between 3 and 8 V [dashed box in (D)]. From the fits, we deduce a lower absolute number of trap states in the nanojunction (gray arrow).

To confirm that Au patch antennas with nanoapertures can work as hole-injecting electrodes, we study hole-only devices with a hole-injecting anode and an electron-blocking cathode, which are expected to provide unipolar hole transport through the organic semiconductor material. The performance of nanojunctions fabricated on the basis of the electrode patches of Fig. 2 is compared to conventional macrojunctions, with an active area of 100 μm by 100 μm. The active area of the nanojunctions (2.4 × 10^−9^ cm^2^) is five orders of magnitude smaller than that of the macrojunction (1.0 × 10^−4^ cm^2^), and edge effects are completely negligible in the macrojunction. The general structure of the hole-only devices and a juxtaposition of macro- and nanojunction properties are displayed in Fig. 3.

*N*,*N*′-di(1-naphthyl)-*N*,*N*′-diphenyl-(1,1′-biphenyl)-4,4′-diamine (NPB; for molecular structure, see Fig. 3A) has been chosen as organic hole transport material for its well-established electronic properties and its use in many standard OLED applications (*50*). The device stack architecture and the flat-band energy landscape are displayed in Fig. 3B. The work function of the polycrystalline Au electrodes is expected to be in the range of −4.4 to −4.7 eV (*51*). The highest occupied molecular orbital (HOMO) level of NPB is at −5.4 eV, and the lowest unoccupied molecular orbital (LUMO) level is at −2.4 eV (*52*). The mismatch of the LUMO level of NPB with the work function of Au makes it feasible to use Au also as an electron-blocking top electrode. 1,4,5,8,9,11-Hexaazatriphenylenehexacarbonitrile (HAT-CN; for molecular structure, see Fig. 3A) is used to functionalize the Au bottom contact. The energy level alignment across the Au/HAT-CN/NPB interface is determined by the Fermi level pinning of the HAT-CN LUMO and the NPB HOMO (*53*). The overall device stack architecture (Fig. 3B) comprises Au(50 nm)/HAT-CN(5 nm)/NPB(135 nm)/Au(140 nm). The principle of operation with an applied voltage exceeding the built-in voltage (*V* > *V*_BI_) is illustrated in Fig. 3C. Hole injection is mediated via the Au patch electrode in conjunction with the HAT-CN interface layer, and injected holes are transported via the NPB HOMO. Electron injection from the top electrode is prevented through the large Schottky barrier between the Au Fermi level and the NPB LUMO.

The effectiveness of the HAT-CN interface functionalization is demonstrated by the current density-voltage (*J*-*V*) characteristics recorded for a macrojunction device with and without HAT-CN functionalization (fig. S5). Upon HAT-CN functionalization, an increase up to six orders of magnitude in hole current at 10 V with a small onset voltage of only 0.9 V for hole injection is observed. Kahn *et al.* (*54*) described the formation of an interfacial dipole of 1.2 eV at the Au/NPB interface, leading to an additional increase in the hole injection barrier. The functionalization by an ultrathin HAT-CN layer, hence, results in a marked reduction of the hole injection barrier due to Fermi level pinning and the related interface dipole, rendering our polycrystalline Au electrodes a superb platform for hole injection in the following device applications.

Figure 3D shows a comparison of the *J*-*V* characteristics of nano- and macrojunctions. The deviation of the current densities at 0 V between the macro- and nanojunction is related to operation of the nanojunction below the instrumental resolution of the source measurement unit of around 1 pA. The estimated onset voltage for hole injection is 1.1 V for the macrojunction and 1.9 V for the nanojunction, respectively. The slight difference can be explained by the larger probability for hole injection and current hotspots in the macroscopic device, as well as by different processing conditions of the Au electrodes, which may alter the work function of the polycrystalline Au surface and the hole injection barrier (*35*, *55*). The nanojunction is stable in a voltage regime between 20 and −20 V, while the macrojunction is only stable between 12 and −12 V exhibiting electrical breakdown for higher voltages. We associate the higher stability of the nanojunction as compared to the macrojunction with the fact that the probability for electrode defects and filament formation is proportional to the device area. It also further signifies the efficiency of the used edge and corner blocking process.

At 10 V, current densities of 1 and 4 A cm^−2^ are reached for the nanojunction and the macrojunction, respectively. The rather small deviation corroborates the cleanliness of the active area after nanoaperture fabrication and oxidative cleaning. The higher current density in the macrojunction likely occurs because of inhomogeneous charge transport via low-ohmic pathways (*56*). As directly visible from fig. S6, the nanojunction operates with an absolute current of only 10 nA at 10 V, which is five orders of magnitude below that of the macrojunction (1 mA).

Efficient hole injection via the Au nanoelectrode is a prerequisite for efficient light emission in OLED structures, where current densities in the range of 1 × 10^−3^ to 1 × 10^−1^ A cm^−2^ at low operating voltages are desirable. The blocking ratio, defined by the current density ratio in forward and reverse bias direction, is 8 × 10^2^ (at 20 V) for the nanojunction in relation to 5 × 10^3^ (at 10 V) for the macrojunction. We attribute the lower blocking ratio for the nanojunction to a slight curvature of the top Au contact induced by the surface topology of the nanoaperture underneath (Figs. 1 and 2, B and D). This is supported by *J*-*V* characteristics of hole-only nanojunctions with varying hole diameters (fig. S7). A smaller nanoaperture diameter is supposed to induce a more pronounced curvature at the top contact, resulting in a higher leakage current translating into a lower blocking ratio in the respective *J*-*V* characteristics. We fit the *J*-*V* characteristics based on the assumption of a space charge–limited current model in combination with a Poole-Frenkel–type charge carrier transport, the latter considering, e.g., trapping and electric field–assisted release of charge carriers within the organic semiconductor (Eq. 1). In Eq. 1, ε_0_ is the vacuum permittivity, ε_r_ is the dielectric constant of NPB, μ_0_ is the zero-field mobility, *V* is the applied voltage corrected for the built-in voltage, *d* is the organic stack thickness, and β is the Poole-Frenkel parameter (*1*, *24*).

$$J=\frac{9}{8}\varepsilon_{0}\varepsilon_{r}\mu_{0}\cdot \frac{V^{2}}{d^{3}}\cdot \text{exp}\left(0.89\cdot \beta \cdot \sqrt{\frac{V}{d}}\right)$$ (1)

**Fig. 4.**
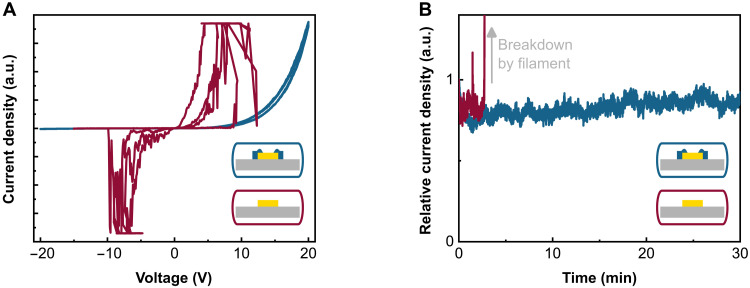
Electrical properties of hole-only devices based on Au patch antennas (1 μm by 1 μm) with (blue lines and inset) and without nanoaperture (red lines and inset). (**A**) Current density–voltage characteristics of two representative junctions with and without nanoaperture. The junctions are voltage cycled three times starting from 0 V. An erratic behavior, likely resulting from filament formation and disruption, is observed for a patch antenna without nanoaperture. (**B**) Constant voltage operation (5 V, dc) of two representative junctions with and without nanoaperture. The device without nanoaperture exhibits breakdown presumably by filament formation after only 3 min of operation, while the device with nanoaperture is stable over the measurement period of 30 min.

With μ_0_ and β as free parameters, the resulting fit functions agree well with the measured *J*-*V* characteristics in a range between 3 and 8 V for both the macrojunction and the nanojunction (Fig. 3E). We restrict the voltage regime to 3 to 8 V to avoid deep-trap filling (low voltages) as well as tunnel injection and Joule heating (high voltages). The zero-field hole mobility amounts to 1 × 10^−5^ cm^2^ V^−1^ s^−1^ for the macrojunction and 3 × 10^−5^ cm^2^ V^−1^ s^−1^ for the nanojunction. The mobility values are in good agreement with literature values of NPB in the presence of trap states (*57*). The Poole-Frenkel parameter β converges to 5 × 10^−3^ for the macrojunction and 2 × 10^−3^ for the nanojunction, respectively. Both the higher hole mobility and the smaller Poole-Frenkel parameter β can be explained by a lower absolute number of trap states and, thus, lower influence on the hole carrier transport within the ultrasmall active volume of the nanojunction. To illustrate the benefits of the nanoaperture concept for the stability and efficiency of devices, we perform a comparative study of the electrical properties for hole-only devices based on Au patch antennas (1 μm by 1 μm) with and without nanoaperture (see Fig. 4).

The *J*-*V* cycles (Fig. 4A) impressively demonstrate the effectiveness of the nanoaperture. While the Au patch antennas with nanoaperture display reliable and deterministic device operation, the ones without nanoaperture show erratic behavior during cycling. The abrupt jumps to the upper current limit are attributed to Au atom migration leading to low-ohmic filament formation, which is incited by the up to threefold electric field enhancement at electrode edges and up to sixfold electric field enhancement at the electrode corners (fig. S1). Upon applying a reverse voltage, the Au filaments are typically disrupted, and the overall current is again dominated by low-ohmic pathways. Our device with nanoaperture electrode does not show any tendency for filament formation and exhibits only a small hysteresis in the *J*-*V* cycles visible in forward bias direction. Considering that the reported *J*-*V* cycles are scaled for comparability, we note that, as expected, the absolute current through the nanoaperture junction is smaller than for the junction without edge insulation.

To further test the device operation stability, we apply a constant dc voltage of 5 V. The time dependence of the relative current density is depicted in Fig. 4B. The device without edge insulation shows electrical breakdown after only 3 min of operation. The relative current density of the nanoaperture device is fully stable over the measurement period of 30 min.

**Fig. 5.**
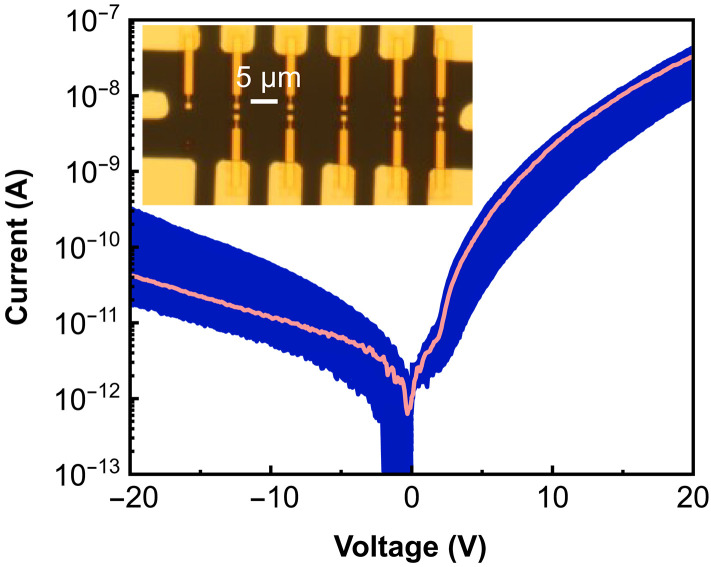
Device reproducibility. The blue area indicates the absolute variation in current-voltage (*I*-*V*) characteristics across 30 individual nanojunctions. At 20 V, the current variation remains within one order of magnitude. The red curve corresponds to the nanojunction shown in Fig. 3. Inset: White-light reflection micrograph of the electrode layout, featuring 11 pixels per block. The vertical and horizontal pixel pitches are 2 and 10 μm, respectively, demonstrating the scalability of the design for display applications. Three such blocks (33 pixels) were fabricated on a single substrate, with only three pixels exhibiting deviation from the standard *J-V* behavior.

We further observe that devices with nanoapertures exhibit an outstanding reproducibility as demonstrated by the *I*-*V* characteristics in Fig. 5, where the blue area marks the variation of the current for 30 individual junctions. The *I*-*V* curve highlighted in red corresponds to the nanojunction discussed in Fig. 3. The current variations remain within one order of magnitude for forward and reverse bias at 20 V, indicating only small variations in the size and depth of the nanoaperture, as well as a high quality of the Au interface after processing. Correspondingly, 91% (30 of 33) of the fabricated nanojunctions show stability against filament formation, again demonstrating the advantages of effective edge coverage and surface quality in nanoaperture devices. Moreover, our encapsulated devices demonstrate sustained functional integrity for at least 14 days under ambient conditions, which highlights the stability of the nanoelectrode structures and metal-organic interfaces. The white-light reflection micrograph (inset in Fig. 5) shows that the pixel fabrication is parallelized and ready for further integration into display-like arrays.

Since the final goal of using Au electrodes is to miniaturize toward resonant plasmonic nanoantennas, we also investigated smaller electrode patches and corresponding apertures. We found that the concept is equally effective for hole-only nanojunctions with a patch antenna size of only 300 nm by 300 nm and a nanoaperture diameter of 200 nm (figs. S4 and S8). The *J*-*V* characteristics (fig. S8A) are comparable to those of the 1 μm–by–1 μm electrode patches. The observed lowering of the blocking ratio is associated with a smaller nanoaperture diameter, without having a negative impact on the remarkable device stability under even harsher dc voltage operation (10 V) over 5.5 hours (fig. S8B). We therefore conclude that our approach is excellently suited for the prospective integration of resonant plasmonic patch antennas with subwavelength dimensions into vertical organic electronic devices.

### Nanoscale OLED pixels

To apply the nanoaperture concept to a light-emitting device, we fabricate nano-OLEDs based on the device concept introduced in Fig. 1. These nanopixels constitute, to the best of our knowledge, the first demonstration of individually addressable subwavelength nano-OLED pixels using a vertical multilayer architecture. By scaling the pixel size down to 300 nm by 300 nm, we enable an efficient light outcoupling via radiative plasmonic modes of the plasmonic patch antenna.

**Fig. 6.**
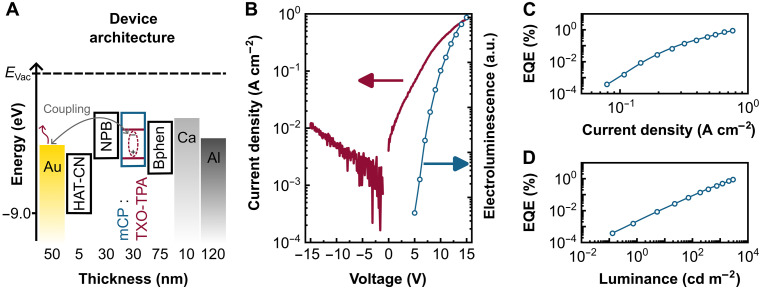
Optoelectronic characterization of a nano-OLED device based on plasmonic Au patch antennas (300 nm by 300 nm) with a 200-nm nanoaperture. (**A**) Flat band energy landscape and device architecture. The stack consists of HAT-CN (5 nm; hole injection layer), NPB (30 nm; hole transport layer), mCP doped with TXO-TPA (30 nm; emissive layer), Bphen (75 nm; electron transport and hole blocking layer), Ca (10 nm; electron injection layer), and Al (120 nm; capping electrode). Light emission is mediated by exciton-plasmon coupling via the Au patch antenna bottom electrode. (**B**) Current density– and electroluminescence (EL)–voltage characteristics recorded between −15 and +15 V. (**C**) EQE as function of the current density for a representative pixel demonstrating EQE values in the percentage regime. (**D**) Luminance characteristics of a representative pixel reaching a peak luminance of ~3000 cd m^−2^, comparable to that of macroscopic OLEDs using the same emitter material. The luminance is calculated considering the 300 nm–by–300 nm patch antenna area.

An overview of the OLED architecture and the basic device properties is provided in Fig. 6. The flat-band energy diagram representing the relevant transport and recombination levels in the device is displayed in Fig. 6A. The bottom anode consists of a 50-nm-thick Au nanoelectrode (300 nm by 300 nm) with a nanoaperture opening (200 nm in diameter). Hole injection is promoted by a 5-nm HAT-CN interface layer, followed by 30 nm of NPB acting as hole transport layer as described above. As light-emitting material, we use the thermally activated delayed fluorescence (TADF) emitter 2-[4-(diphenylamino)phenyl]-10,10-dioxide-9*H*-thioxanthen-9-one (TXO-TPA) embedded in 1,3-bis(*N*-carbazolyl)benzene (mCP) as host material (7 vol %), which constitutes an efficient and commonly used host-emitter combination (*58*). The emissive layer (30 nm) is separated by a 75-nm bathophenanthroline (Bphen) electron transport layer from the top electrode. The flat electron-injecting top cathode consists of 10-nm Ca and 120-nm Al. The asymmetric device architecture is intended to achieve exciton recombination far away from the top cathode to avoid quenching by the cathode layers. Excitons generated within the emissive layer efficiently couple to the radiative plasmonic mode(s) of the plasmonic Au patch antenna electrode, enabling far-field radiation through the glass substrate.

The fabricated nano-OLEDs exhibit stable operation under both forward and reverse bias, with a pronounced *J*-*V* asymmetry (Fig. 6B). Light emission begins at 5 V, and the recorded electroluminescence (EL) intensity increases with applied voltage (Fig. 6B). Consistent with previous results on nanoaperture-based hole-only devices, no signs of filament formation, subsequent short circuits, or device failure are observed, highlighting the robustness and inherent stability of the nanoaperture concept for devices based on individually addressable nanoscale electrode patches. The pixels can be reliably cycled under forward and reverse bias without failure (see fig. S9A). We achieve remarkably high EQEs of up to 1% (Fig. 6C), with minimal pixel-to-pixel variation (see fig. S9B). An EQE in the percentage range indicates a high internal recombination efficiency, as expected from the high-performance material stack in combination with the nanoaperture, which ensures balanced charge carrier injection even at the nanoscale. In contrast, nano-OLEDs without nanoapertures exhibit instability due to metallic filament growth or unbalanced injection via electrode edges and corners, resulting in substantially lower EQEs (10^−4^ to 10^−2^%).

Notably, the nano-OLED device achieves an exceptional maximum luminance of ~3000 cd m^−2^, which is not yet saturated and matches the performance of macroscopic devices using the same emitter material (*58*). The dynamic response of the nano-OLED pixel was further characterized by transient EL (fig. S10). When driven by a 9-V square-wave pulse (10% duty cycle) at 1 kHz, well above standard video frame rates, the device shows rapid switching dynamics, with a rise time of 50 μs and a fall time of 100 μs.

These results confirm the scalability and application potential of the nanoaperture concept for next-generation nano-optoelectronic devices. Both the high EQE and elevated luminance are governed by the plasmon-enhanced emission mechanism discussed in the following section.

**Fig. 7.**
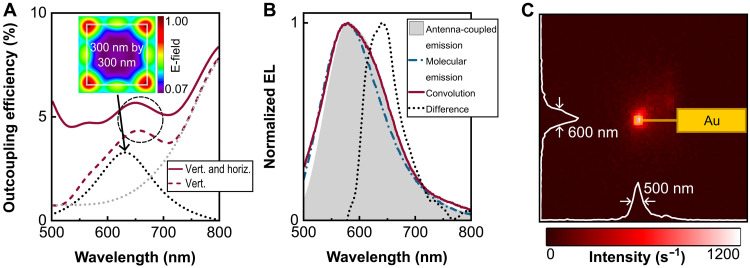
Nano-OLED light emission mediated by plasmonic modes of the Au patch antenna (300 nm by 300 nm). (**A**) Simulated light outcoupling efficiency of the nano-OLED stack, averaged over multiple laterally distributed point dipole positions within the 200-nm nanoaperture. Dipoles are placed 50 nm above the Au patch. The solid curve shows the combined contribution from horizontally and vertically oriented dipoles, while the dashed curve isolates the vertical dipole contribution, illustrating coupling to plasmonic antenna modes. The inset displays the simulated electric field distribution of the dominant quadrupolar-like n_22_ mode at 650 nm, calculated 15 nm above the Au surface. The n_22_ resonance (black dotted line) and contributions from lower-order modes (n_21_, n_11_, and n_10_; gray dotted line) are indicated as guides to the eye. (**B**) Normalized EL spectrum measured at 15 V (gray; antenna-coupled emission). The convolution of a macroscopic standard OLED spectrum (molecular emission) with the simulated outcoupling efficiency spectrum [from (A)] closely resembles the nano-OLED spectrum, confirming predominant coupling to the n_22_ plasmonic mode. The spectral shaping is highlighted by the normalized difference spectrum of antenna-coupled emission and molecular emission. (**C**) Spatial emission map from a representative nano-OLED pixel. The pixel area and electrical contact lines are overlaid in yellow. Emission is centered within the nanoaperture-defined pixel. Linecuts along the vertical and horizontal axes reveal a point-like emission pattern with a FWHM below 600 nm, limited by the microscope resolution.

Last, we analyze the light emission properties of the nano-OLED pixel (see Fig. 7). To quantify its light outcoupling efficiency, we perform electromagnetic simulations using the finite-difference time-domain (FDTD) method (Ansys Lumerical), based on a simplified device stack as depicted in fig. S11. The simulations use electric dipoles with both vertical and horizontal orientations, representing the statistically distributed excitonic emitter orientations within the emissive layer. These dipoles excite plasmonic patch antenna modes confined within the 200-nm-diameter nanoaperture. To account for the contributions of the full range of Au patch antenna modes, we include lateral spatial averaging of the dipole positions. Vertical dipoles predominantly couple to the dominant n_22_ mode at 650 nm (Fig. 7A, inset field distribution and black dotted line), while lower-order modes (n_21_, n_11_, and n_10_; see fig. S12) contribute off-resonantly via spectral tailing (see Fig. 7A, gray dotted line). The combined contributions from horizontally and vertically oriented dipoles result in an overall light outcoupling efficiency in the ~5% range (Fig. 7A, solid line), in agreement with the experimentally measured EQE. In Fig. 7B, we compare the EL spectrum of the nano-OLED pixel with that of a standard macroscopic OLED pixel (pixel area, 3 mm^2^) using transparent indium tin oxide anodes. The convolution of the standard OLED spectrum (molecular emission) with the simulated outcoupling efficiency spectrum (solid line in Fig. 7A) closely matches the measured nano-OLED spectrum (antenna-coupled emission). The spectral shaping through plasmonic antenna effects is highlighted by the difference spectrum of antenna-coupled emission and molecular emission. To further substantiate the plasmonic antenna effect in our nano-OLEDs, we compared their EL spectra with those of micro-OLEDs based on 2 μm–by–2 μm Au electrodes (see fig. S13). While the micro-OLED spectrum closely matches the macroscopic OLED spectrum due to red detuning of plasmonic antenna effects, the nano-OLEDs show pronounced spectral shaping above 600 nm through the 300 nm–by–300 nm Au patch antennas. This confirms that the spectral shaping of the TXO-TPA emission arises from the coupling to the plasmonic modes, particularly the dominant n_22_ mode at 650 nm. Figure 7C shows the spatial EL distribution of the nano-OLED pixel, with the actual pixel area and electrical contacts highlighted in yellow. From intensity line cuts, we determine a full width at half maximum (FWHM) of the emission spot below 600 nm, limited by the optical resolution of the microscope. These results underscore the potential of the plasmonic patch antenna electrode concept for highly integrated OLED display technologies.

## DISCUSSION

We identify the inhomogeneous three-dimensional electric field distribution at nanoelectrodes as the key factor responsible for spatially unbalanced charge carrier injection, inefficient transport and recombination, and device failure due to filament formation in vertical nano-optoelectronic architectures. To mitigate these detrimental scaling effects, we introduce a concept that uses strategically engineered insulating layers to selectively passivate the edges and corners of metallic nanoelectrodes, while leaving a nanoscale aperture at the flat electrode surface. The incorporation of such a nanoaperture proves essential for ensuring reliable optoelectronic device characteristics and long-term stability. By confining charge injection to a planar interface with a homogeneous electric field distribution, erratic *J*-*V* characteristics, commonly observed in hole-only devices with bare Au nanoelectrodes (1 μm by 1 μm) due to edge-induced filament formation, can be entirely suppressed. Our nanofabrication process is highly reproducible, yielding operational pixels with over 90% success rate, and thus enables the realization of organic (opto)electronic devices with individually addressable nanoscale electrodes. In hole-only configurations, Au nanoelectrodes featuring nanoapertures and HAT-CN surface functionalization exhibit low turn-on voltages down to 1.9 V and exceptional stability up to 20 V, outperforming their 100 μm–by–100 μm macroscopic counterparts.

We have integrated patch antennas with these nanoapertures into a vertical nano-OLED architecture with individually addressable pixels measuring 300 nm by 300 nm, to the best of our knowledge, the smallest individually addressable pixel size reported for OLEDs to date. These nano-OLEDs exhibit moderate turn-on voltages (~5 V), stable operation over a wide bias range (−15 to +15 V) and time, and fast transient responses exceeding standard video frame rates. Light emission is governed by coupling of excitonic states to plasmonic modes of the Au nanopatch antenna, as supported by both electromagnetic simulations and the spectrally shaped EL observed experimentally. This plasmon-mediated emission mechanism enables EQEs of up to 1%, spatial localization of the emission to the nanoscale pixel area, and luminance levels reaching 3000 cd m^−2^. Realizing display applications based on this concept requires coordinated optimization of the organic stack, patch antenna electrode design, and precision nanofabrication. First, in the current prototype stack, the EQE approaches saturation at relatively high voltages. This behavior arises from space charge accumulation and imbalanced charge carrier transport. Holes are injected efficiently, while electrons require higher bias and must traverse a thick (75 nm), low-mobility transport layer, leading to charge carrier accumulation at interfaces. This leads to higher operating voltages to compensate for the internal electric fields and to balance recombination. These effects originate from the prototype stack design rather than from a fundamental limitation of our nano-OLED concept. The Au patch antenna electrodes already enable efficient hole injection with an onset below 2 V. Incorporating optimized p-i-n OLED architectures with doped transport layers and specific confinement layers, as established in commercial displays, is expected to reduce operation voltages to ~5 V and further enhance EQE to meet modern display requirements. Second, the implementation of tailored antenna designs will enable even more efficient coupling between excitonic emitters and radiative plasmonic modes (*59*) to enhance both vertical and horizontal dipole emission. This will likely improve the outcoupling efficiency by up to an order of magnitude and allow for strong spectral shaping. Third, these advances must be compatible with ultrahigh-density pixel integration (e.g., >10,000 pixels per inch), made possible by advanced lithographic techniques (*60*). However, scaling to these densities introduces challenges such as electrical and optical cross-talk between adjacent pixels (*61*), necessitating careful codesign of antenna structures, organic stack architecture, and nanomanufacturing protocols. A unified approach to these challenges will unlock breakthroughs in ultrahigh-resolution nanodisplays, on-chip photonic integration, and next-generation nano-optoelectronic devices.

## MATERIALS AND METHODS

### Fabrication of hole-only macrojunctions

Glass substrates (Karl Hecht; thickness, 170 μm) were thoroughly cleaned by sequential ultrasonication (15 min each) in double-distilled water (CarlRoth) with Mucasol detergent (schuelke), in double-distilled water, in acetone, and in isopropanol, followed by drying in a nitrogen stream. The active area of the junction was defined by the overlap of perpendicular bottom and top contact stripes. The width of the stripes was defined to 100 μm each by stainless steel shadow masks (Beta LAYOUT GmbH), resulting in a junction area of 100 by 100 μm^2^.

The metal contacts and organic films were deposited in two separate vacuum chambers to avoid contamination of the organic materials. In both chambers, the deposition rate and thickness were monitored using a quartz crystal microbalance. Au was purchased at a purity of 99.99% and evaporated from commercial molybdenum boat sources (Kurt J. Lesker) at a base pressure below 3 × 10^−6^ mbar and at a deposition rate of 1.5 nm s^−1^. The Au bottom contact was deposited with a thickness of 50 nm. During transfer to the organic chamber, the bottom contact was briefly exposed to ambient conditions under yellow light for 5 to 10 min.

The organic materials were evaporated from boron nitride crucibles by resistive heating at a base pressure of 10^−9^ mbar. HAT-CN and NPB were purchased from Ossila at double sublimed and sublimed grade, respectively, and used without further purification. Both materials were deposited through a large area shadow mask widely covering the bottom contact. Five-nanometer HAT-CN was deposited at a deposition rate of 2 nm min^−1^, and 135-nm NPB was deposited at a rate of 8 nm min^−1^. For the reference device without HAT-CN, 140-nm NPB was deposited.

After ambient exposure for 10 min during sample transfer, the Au top contact was deposited in the metal chamber with a thickness of 140 nm. Last, the devices were encapsulated in a glovebox [Jacomex; 0.0 parts per million (ppm) O_2_ and 0.0 ppm H_2_O] with a glass slide and epoxy resin (LOCTITE EA9492 LI) to prevent penetration of oxygen and water during measurements.

### Fabrication of hole-only nanojunctions and nano-OLEDs

The electrode layout was fabricated using optical lithography (AR-U4030, Allresist GmbH). After development, the samples were rinsed with water, and were plasma cleaned [200 W, 60 s, 10 standard cubic centimeters per minute (SCCM) oxygen]. A 10-nm chromium adhesion layer followed by a 70-nm Au layer was deposited via electron beam physical vapor deposition at a rate of 1 nm s^−1^. The photomask was then removed using a liftoff process in acetone. The patterned substrates were further cleaned by sequential ultrasonication for 10 min each in deionized water, acetone, and isopropanol. To ensure a residue-free surface, oxygen plasma cleaning was performed for 10 min (power, 250 W; oxygen flow, 20 SCCM).

For high-resolution EBL, a double-layer resist consisting of 100-nm PMMA 600K and 20-nm PMMA 950K (Allresist GmbH) was spin coated onto the cleaned substrate and baked at 150°C for 3 min. A conductive resist (Electra 92, Allresist GmbH) was then spin coated on top to serve as a discharge layer. Electron-beam patterning was carried out using a Zeiss SEM Gemini 450 operated at 30-kV acceleration voltage and 35-pA beam current. The typical exposure dose for the antenna pattern was ~800 μC cm^−2^.

The development process was performed at room temperature as follows:

1) Immersion in water for 20 s to remove the conductive resist

2) Soaking in a 3:1 methyl isobutyl ketone:isopropanol mixture for 60 s to develop the PMMA pattern

3) Rinsing in isopropanol for 30 s to stop development

4) Drying with a nitrogen stream

A 50-nm Au layer was then thermally evaporated at a deposition rate of 1.5 nm s^−1^ at a base pressure below 3 × 10^−6^ mbar. Liftoff was completed by overnight soaking in acetone (pro analysis grade), followed by 10 s of ultrasonic agitation. To create a planarization layer, 100 nm of HSQ (SX AR-N 8200, Allresist GmbH) was spin coated along with a conductive resist. The HSQ layer was structured using a second EBL step. A gradient dose ranging from 0 to 100% of 2250 μC cm^−2^ was applied for the antenna patch, and a constant dose of 2250 μC cm^−2^ for the remaining insulating layer. Development was performed using a 1% TMAH solution in water at room temperature. An AFM scan was used to verify the development quality of the antenna structures.

To prevent direct or parasitic electrical contact between the bottom Au connector and the top metal electrode, a thick (~800 nm) PMMA layer was spin coated. A third EBL step was then performed to open a window aligned with the antenna region, defining the pixel. Following this step, oxidative cleaning was performed using highly diluted Lugol’s solution (1:2000) for 8 to 10 s, followed by rinsing in water.

For organic layer deposition, a stainless-steel shadow mask (Beta LAYOUT GmbH) with an aperture of 140 μm by 100 μm was aligned on the nanoelectrode array. A second shadow mask with a slightly larger opening (200 μm by 120 μm) was aligned after organic deposition for the top contact. Masks were fixed using tension strings, and alignment required ~45 min of ambient exposure. All subsequent deposition steps were performed as described for the macrojunction.

mCP was purchased from Ossila at unsublimed grade and purified by gradient sublimation. TXO-TPA was purchased from Lumtec at sublimed grade and used without further purification as the TADF emitter. Bphen (spectrophotometric grade, >99.0%) was obtained from Sigma-Aldrich and used as received. Five-nanometer HAT-CN was deposited at 2 nm min^−1^, followed by 30-nm NPB at 4 nm min^−1^. The emissive layer (30 nm) was coevaporated from separate crucibles containing mCP and TXO-TPA. The deposition rate ratio was adjusted to achieve 7 vol % TXO-TPA doping, with a total deposition rate of 7 nm min^−1^. Last, 75 nm of Bphen were deposited at a rate of 6 nm min^−1^.

Following organic layer deposition, the second shadow mask was aligned as described above. Top contact deposition was performed in a glovebox-integrated system (Leybold). A 10-nm Ca layer was deposited at 0.2 to 0.3 Å s^−1^, followed by 120-nm Al at 2 to 3 Å s^−1^, while rotating the sample at 16 rpm. The devices were then encapsulated inside the glovebox, without further exposure to ambient conditions, using the same procedure described previously. For better visualization, a simplified process schematic is shown in fig. S14.

### Atomic force microscopy

AFM topography images were recorded in tapping mode at a Veeco Dimension Icon using standard tapping mode AFM probes (NanoWorld, NCHR; 320 kHz, 42 N m^−1^). Conductive AFM images were recorded using the same instrument in contact mode with the TUNA application module and conductive AFM probes (NanoWorld, SCM-PIC; 13 kHz, 0.2 N m^−1^). The investigated electrodes were grounded, and a bias voltage of 100 mV was applied. Data analysis was carried out in Gwyddion (*62*).

### (Opto)electronic device characterization

Current density–voltage characteristics of hole-only macrojunctions were recorded with a B1500A semiconductor parameter analyzer (Keysight Technologies). Contact was established through tungsten probe needles (FormFactor Inc.), which were mounted to triaxial probe arms (FormFactor Inc.) and micromanipulators (DPP210, FormFactor Inc.). In case of the nanojunctions, voltages were applied by a source meter unit Keithley 2636B (Keithley Instruments Inc.). Contact was established through copper-beryllium probe needles (Semprex Corp.), which were mounted to triaxial probe arms (Cascade Microtech) and micromanipulators (DPP 220, Cascade Microtech). The current density–voltage characteristics of hole-only nanojunctions were measured in voltage steps of 100 mV with an integration time of 20 ms. The source limit was set to 100 nA. EL of the nano-OLED device was recorded via an oil-immersion microscope objective [Plan-Apochromat, 100×, numerical aperture (NA) = 1.45; Nikon) and analyzed by a spectrometer (Shamrock 303i, 80 lines mm^−1^, blazing at 600 nm or mirror) equipped with an electron-multiplied charge-coupled device (iXon A-DU897-DC-BVF, Andor, EM gain 100). Source meter unit and electron-multiplied charge-coupled device camera were synchronized by a LabVIEW program to allow for correlated data analysis. Consecutive constant voltage steps were applied for measuring the voltage-dependent spectra. Typical integration times for recording the spectra ranged from 0.1 s to 1 s. The EQE was calculated from the EL spectra considering the detection efficiency of the optical setup. The detection efficiency of the setup is depicted in fig. S15. The luminance was calculated according to the International Commission on Illumination (CIE). First, the radiant power (in watts per nanometer) was computed from the detected photon intensity weighted by the area normalized EL spectrum. The luminous flux (in lumens) was then obtained by integrating the radiant power weighted with the luminous efficacy at 555 nm (CIE standard, 683 lm W^−1^) and the photopic luminous efficiency function. Last, the luminance (in candela per square meter) was derived under the assumption of Lambertian emission into a hemisphere, normalized to the patch antenna area of 300 nm by 300 nm.

### Transient EL characterization

The nano-OLED pixel was electrically driven using a square wave voltage signal generated by a function generator (DS345, Stanford Research Systems). The waveform was configured with a frequency of 1 kHz, a peak-to-peak amplitude from −1 to 9 V, and a duty cycle of 10% (9-V “on” state duration, 100 μs per cycle). A synchronization output from the function generator served as the reference start signal for the time-to-digital converter (quTAU; time resolution, 80 ps). EL from the nano-OLED pixel was collected using an oil-immersion objective (Plan-Apochromat, 100×, NA = 1.45; Nikon) and focused onto a single-photon avalanche photodiode (APD; SPCM-AQR-13, Excelitas). The resulting transistor-transistor logic pulses from the APD were used as stop signals for the time-correlated single-photon counting measurement. Time delays between start and stop signals were recorded by the quTAU, generating a histogram with a bin width of 80 ns.

The macroscopic reference OLED was characterized using a similar home-built setup. The OLED pixels were contacted using micromanipulators (DPP210, FormFactor Inc.), shielded triaxial probe arms (100525, FormFactor Inc.), and tungsten needles (PTT-120, FormFactor Inc.). EL was detected using a photomultiplier tube (PMT) in combination with a collection lens. The PMT output signal was amplified using a current amplifier (DHPCA-100, high-speed mode, amplification: 10^3^) and monitored via an oscilloscope (TDS 3032, Tektronix) at 50-ohm input impedance. The OLED pixel was biased using a pulse generator (Hewlett Packard), with synchronization between the voltage waveform and the EL signal established via a second oscilloscope input set to 1-megohm input impedance.

### Numerical simulations

Three-dimensional FDTD simulations were carried out using the Maxwell equation solver in the commercial software Ansys Lumerical. A high-resolution mesh (1 nm in all dimensions) was applied to the plasmonic nanopatch region to ensure accurate field resolution. Perfectly matched layers (PMLs) were implemented at all simulation boundaries to eliminate artificial back reflections. To approximate an infinite geometry, the top cathode, organic stack, and glass substrate were extended into the PML regions. The Au electrical connector was modeled as a semi-infinite structure using the PML boundary condition. An electric dipole point source was used as the excitation source in all simulations. For enhanced mode visualization, the mode field distribution was simulated using an Au nanopatch without an electrical connector. In contrast, the light outcoupling simulations were performed using a connected Au nanopatch structure (see fig. S11).

## Supplementary Materials

### This PDF file includes:

Sections S1 to S11 Figs. S1 to S15 References
