# Supplementary material for: Individually addressable nanoscale OLEDs
Source: Sci Adv. 2025 Oct 22;11(43):eadz8579. doi: 10.1126/sciadv.adz8579 (PMC12542931; doi:10.1126/sciadv.adz8579)
Supplement: Supplementary file 1 — Sections S1 to S11 Figs. S1 to S15 References [file sciadv.adz8579_sm.pdf]

Science Advances
AAAS

Supplementary Materials for

Individually addressable nanoscale OLEDs

Cheng Zhang *et al.*

Corresponding author: Cheng Zhang, cheng.zhang@uni-wuerzburg.de; Bert Hecht, bert.hecht@uni-wuerzburg.de; Björn Ewald, bjoern.ewald@uni-wuerzburg.de; Jens Pflaum, jpflaum@physik.uni-wuerzburg.de

Sci. Adv. **11**, eadz8579 (2025)
DOI: 10.1126/sciadv.adz8579

This PDF file includes:

Sections S1 to S11
Figs. S1 to S15
References

SI 1: Electrostatic Simulations

When a macroscopic extended electrode (in our case anode) is scaled down to a nanoscale electrode with subwavelength dimensions, electric field inhomogeneities will dominate the overall device operation in a vertical device architecture. As sketched in Fig. S1A, the static electric field is expected to be locally intensified at the edges of the electrodes when a DC voltage bias is applied, whereas uniform field lines are expected to be found at the extended top planar electrode. An electrostatic simulation was performed using COMSOL Multiphysics 6.0, AC/DC module, to demonstrate this effect, as depicted in Fig.S1B and C. The electric field distribution beneath the extended planar top cathode is indeed uniform at 1 nm distance, but there is a notably enhanced inhomogeneous electric field at the corners (> 6 times enhancement) and edges (> 3 times enhancement) of the structured bottom electrode, compared to the planar top electrode. Such electric field inhomogeneities lead to localized edge-induced charge injection and enhanced electromigration causing filament growth.

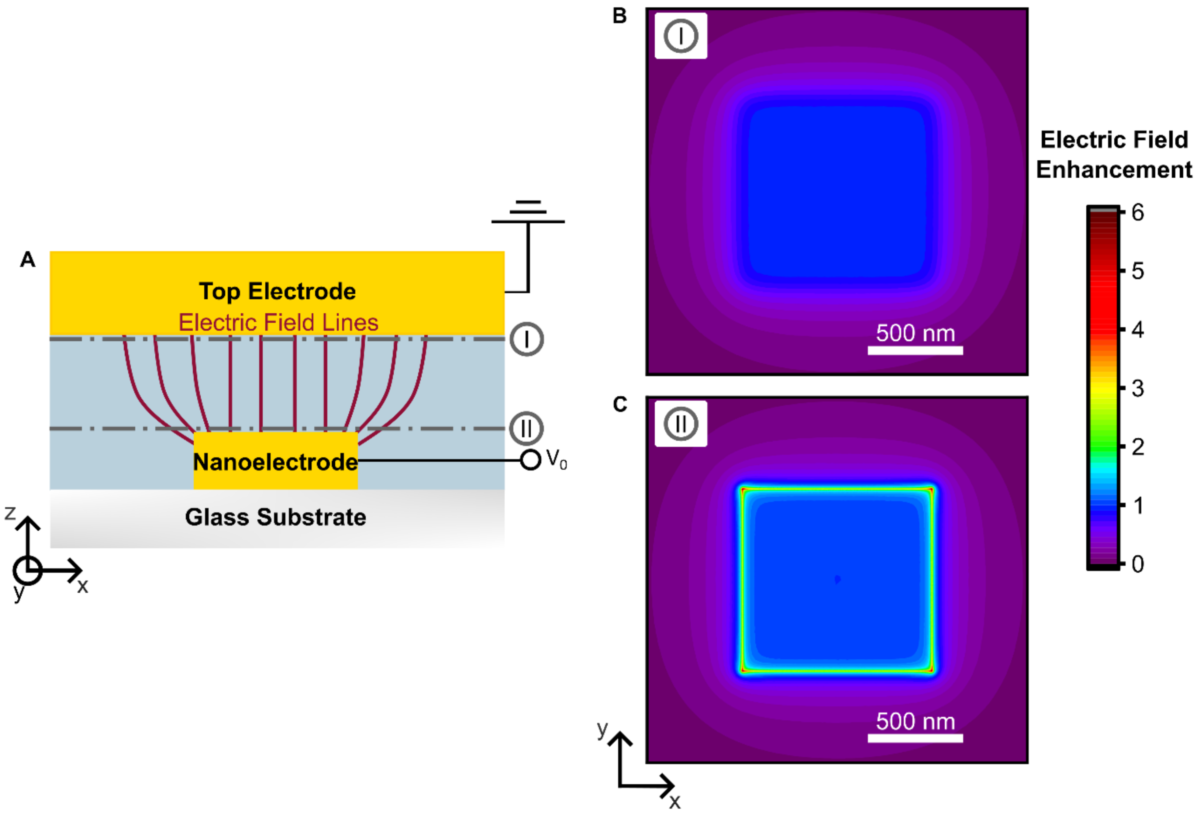

Fig. S1. Influence of electric field inhomogeneity in a vertical organic optoelectronic device using a structured bottom electrode. (**A**) Sketch of the electrostatic field distribution in a vertical metal-organic-metal device. The structured Au anode is voltage biased, while the flat Au electrode is grounded. (**B**) Electrostatic field distribution 1 nm below the flat extended Au cathode and (**C**) 1 nm above the 1 × 1 μm^2 structured Au anode (both plains marked by dashed dotted lines in A)). To obtain a realistic estimate of the local field enhancement all corners and edges are rounded with a 10 nm radius of curvature. The distance between two electrodes is 140 nm. The organic layer between the electrodes is modelled by a dielectric (relative permittivity $\varepsilon_r = 2.25$). The refractive index of solidified HSQ (1.4) matches the refractive index of the organic materials (about 1.5). The HSQ nanoaperture will thus not influence the electric field distribution (*63*). A voltage of 10 V is applied between the electrodes. The absolute electric field is normalized to the bottom electrode center.

SI 2: Morphology and Structure of Au Bottom Electrodes

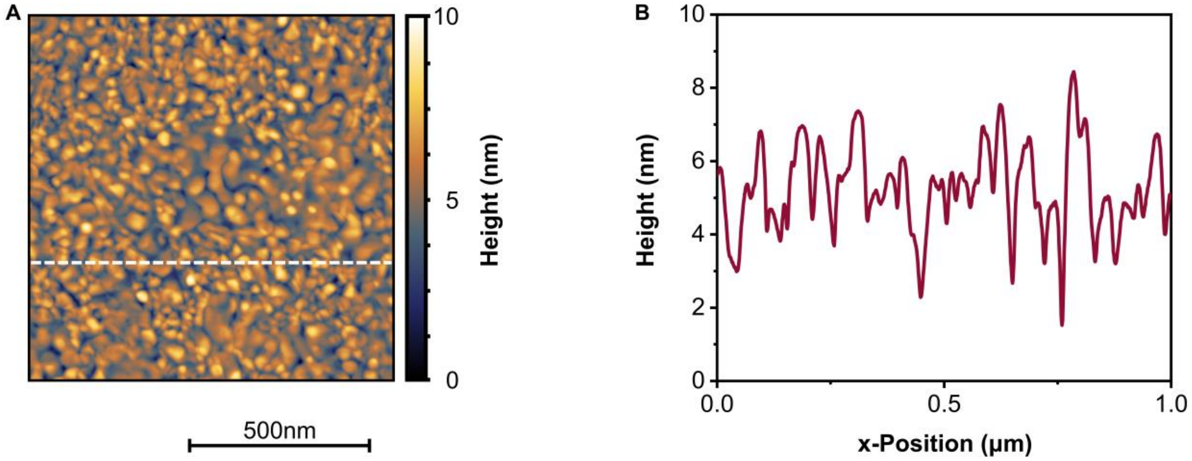

Fig. S2. Morphology of Au thin films (thickness 50 nm) deposited at a moderate rate of 1.5 nm·s^{-1}. (**A**) Tapping mode AFM image of a representative Au film area (1 x 1 µm^2) with a root mean square (rms) roughness of 1 nm and an average roughness of 5 nm. (**B**) Height cross section along the white dashed line in A.

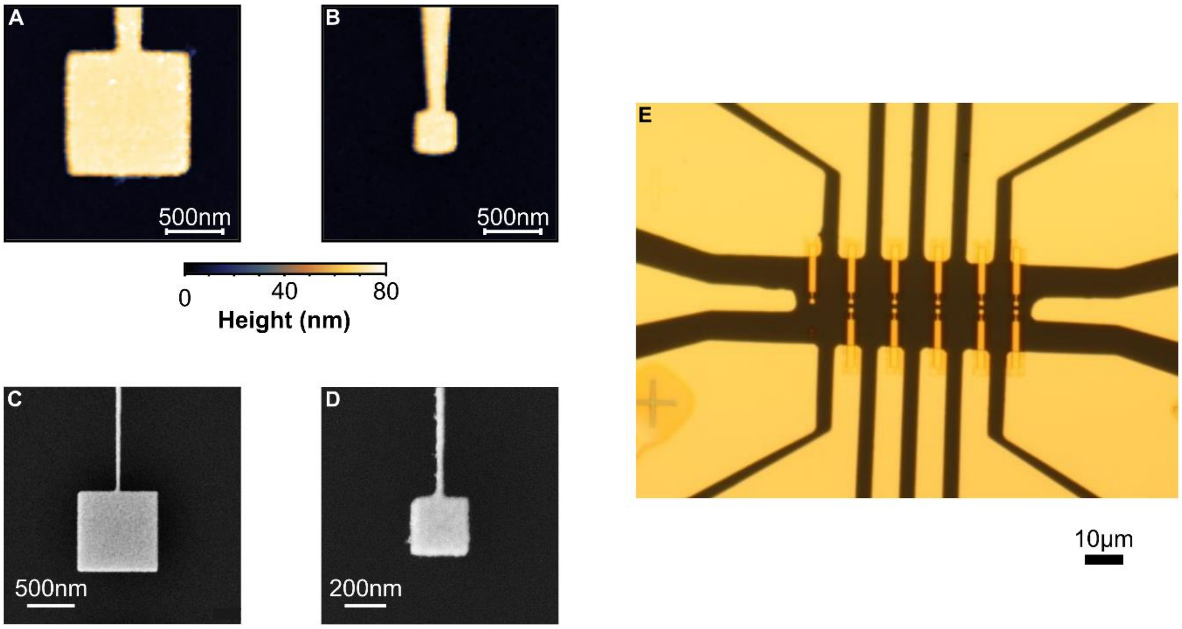

Fig. S3. Characterization of Au patch antennas with electrical connectors after lift-off and before applying the nanoaperture. (**A**), (**B**) Tapping-mode AFM images of a 1 x 1 μm^2 and of a 300 x 300 nm^2 Au patch antenna, respectively. (**C**), (**D**) SEM micrographs (Everhart-Thornley-detector, acceleration voltage 10 kV) of a 1 x 1 μm^2 and a 300 x 300 nm^2 Au patch antenna, respectively. Note the well-defined surface and edges of the patch antennas fabricated by EBL and thermal evaporation of Au with only tiny residuals left at the edge. (**E**) White-light reflection micrograph of the electrode layout with 11 individually addressable pixels (1 x 1 μm^2).

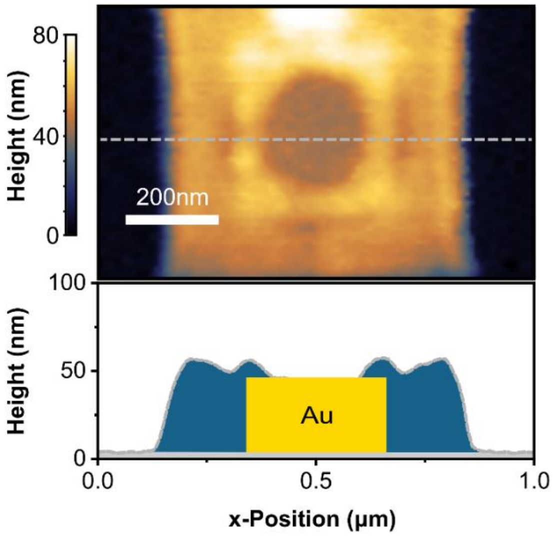

Fig. S4. AFM analysis of an Au patch antenna with nanoaperture. Top: Tapping-mode AFM image of a 300 x 300 nm^2 Au electrode with electrical connector. The electrode edges and the connector are fully covered by HSQ. A central 200 nm diameter nanoaperture in the HSQ provides access to the Au patch antenna. Bottom: Cross sectional height profile along the cut indicated by the dashed grey line (top).

SI 3: HAT-CN Functionalization of Au Anodes

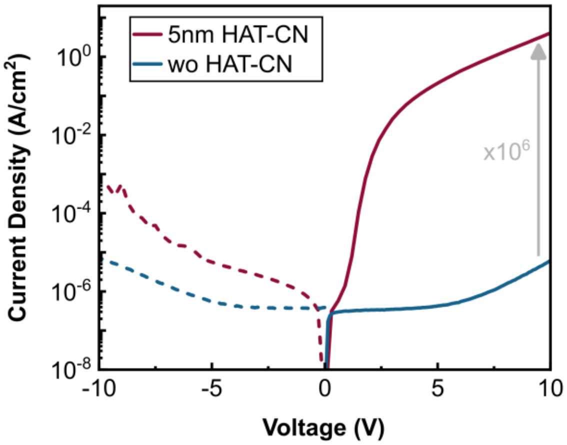

Fig. S5. Current density-voltage characteristics of hole-only macrojunctions (100 x 100 µm²) with 5 nm HAT-CN functionalization and without HAT-CN functionalization. The hole current density at 10 V is increased by 6 orders of magnitude upon HAT-CN functionalization. The device architectures are 50 nm Au / 5 nm HAT-CN / 135 nm NPB / 140 nm Au and 50 nm Au / 140 nm NPB / 140 nm Au, respectively.

SI 4: Absolute Current in Hole-Only Macro- and Nanojunctions

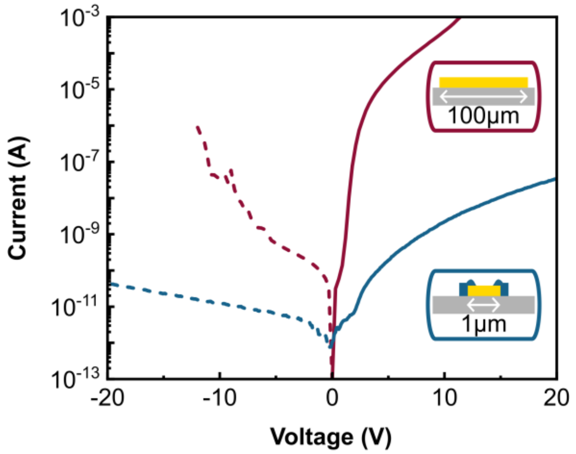

Fig. S6. Current-voltage characteristics of a hole-only macrojunction (electrode patch: 100 x 100 µm^2, active area: 1.0·10^{-4} cm^2) and nanojunction (electrode patch: 1 x 1 µm^2, nanoaperture diameter: 550 nm, active area: 2.4·10^{-9} cm^2) in semilogarithmic presentation. The absolute current at 10 V is 5 orders of magnitude smaller in the nanojunction (10 nA) compared to the macrojunction (1 mA).

SI 5: Influence of Nanoaperture Diameter on the Blocking Ratio

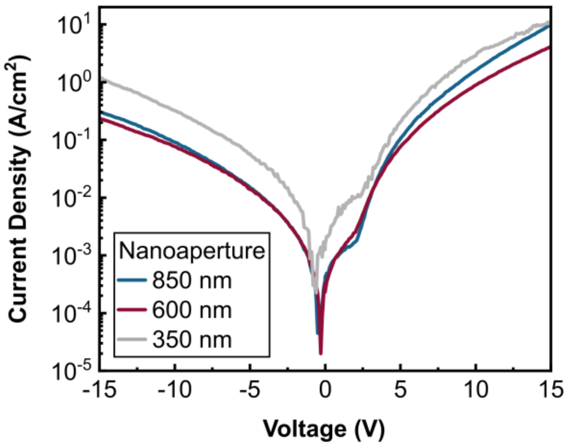

Fig. S7. Current density-voltage characteristics of three hole-only nanojunctions (electrode patch: 1 x 1 µm^2) with varying nanoaperture diameter (850 nm, 600 nm, 350 nm) and a fixed depth of 50 nm in semilogarithmic presentation. The blocking ratio @15 V (forward to reverse current density) decreases with decreasing nanoaperture diameter from 33 (850 nm) to 17 (600 nm) and finally to 8 (350 nm). We attribute this effect to a slight increase in the top contact curvature imposed by the nanoaperture shape.

SI 6: Hole-Only Nanojunctions with 300 x 300 nm^2 Au Nanoelectrodes

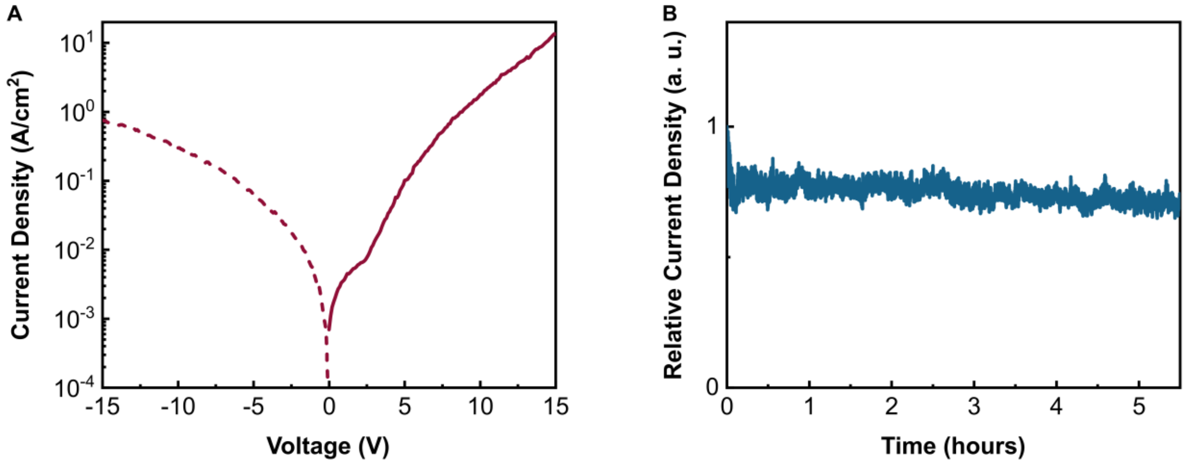

Fig. S8. Hole-only nanojunctions with an electrode size of 300 x 300 nm^2 and a nanoaperture opening of 200 nm in diameter. (**A**) Current density-voltage characteristics of a representative nanojunction pixel in semilogarithmic presentation. (**B**) Constant voltage operation (10 V, dc) of the nanojunction. The current density is stable over the observation period of 5.5 hours and no tendency for filament formation is observed.

SI 7: Stability and External Quantum Efficiency of Nano-OLEDs

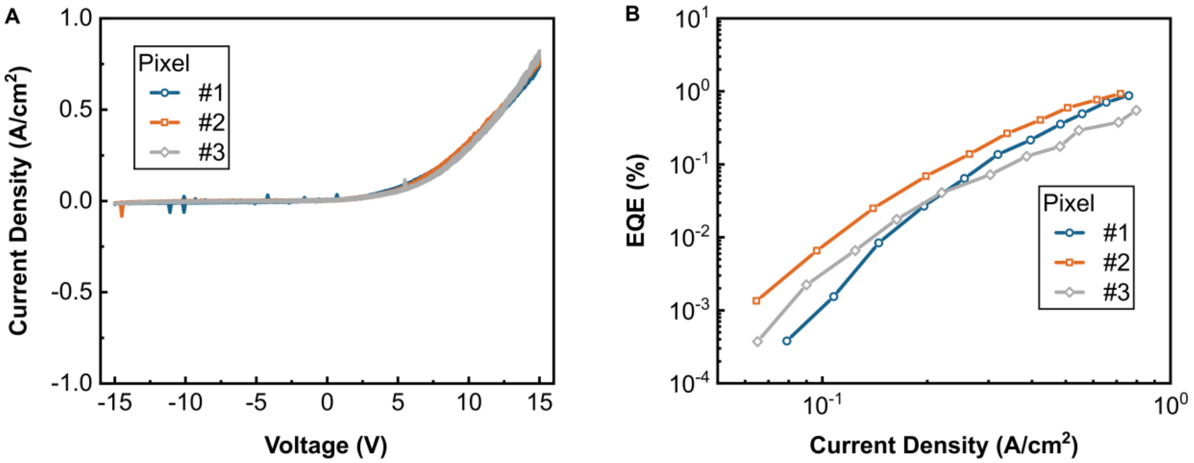

Fig. S9. Stability and external quantum efficiency of three representative nano-OLED pixels. (**A**) Current density-voltage characteristics from -15 V to 15 V. The pixels have been cycled three times forth and back starting from 0 V without any tendency for device failure. (**B**) External quantum efficiencies (EQEs) as function of the current density with only a small variation between the pixels.

SI 8: Transient Response of Nano-OLEDs

We have investigated the dynamic response of our nano-OLED device by applying a voltage duty cycle (9 V (100 µs) / -1 V (900 µs)) to the nano-OLED (300 x 300 nm^2). The EL response was tracked by time-correlated single photon counting. We have also measured a macroscopic standard-OLED (ITO/PEDOT:PSS (40 nm)/NPB (30 nm)/mCP:TXO-TPA (30 nm)/3TPYMB (50 nm)/LiF(1 nm)/Al(120 nm)) with an active pixel area of 3 mm^2. In this case the transient EL response was tracked by a photomultiplier tube. Respective data are shown in Fig. S10. The transient response of our nano-OLED is more than sufficient to support video frame rates for display applications (> 60 frames per second) and widely resembles the transient response of the standard-OLED despite a parasitic overlap capacitance induced by the large area top contact. The rise time is 50 µs (90 %) and the fall time is 100 µs (10 %).

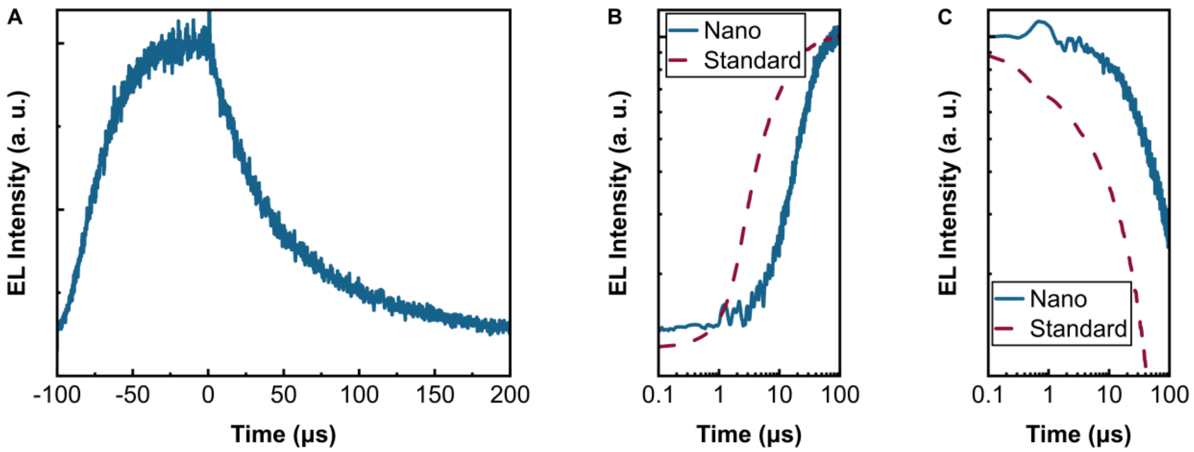

Fig. S10. Transient EL response of a nano-OLED device based on Au nanoelectrodes (300 x 300 nm^2) with a 200 nm diameter nanoaperture. A duty cycle of 9 V (100 µs) / -1 V (900 µs) was applied. (**A**) Transient EL response over a time frame of 300 µs. (**B**) Double-logarithmic presentation of the EL response upon switching on (9 V) the device. (**C**) Double-logarithmic presentation of the EL response upon switching off (-1 V) the device. The transient response of a standard ITO reference OLED based on the same emitter material is displayed for comparison (dashed red lines in B and C). The transient dynamics is more than sufficient to support video frame rates (> 60 frames per second). The RC time constant is not reduced in case of the nano-OLED as the overall circuit capacitance (dielectrics in combination with extended top electrode) dominates the device response, especially visible in the slightly slower response upon switching on the device. Overall, the dynamic response of the nano-OLED resembles the response of the standard OLED with a rise time of 50 µs (90 %) and a fall time of 100 µs (10 %).

SI 9: Plasmonic Mode Analysis of the Au Patch Antenna

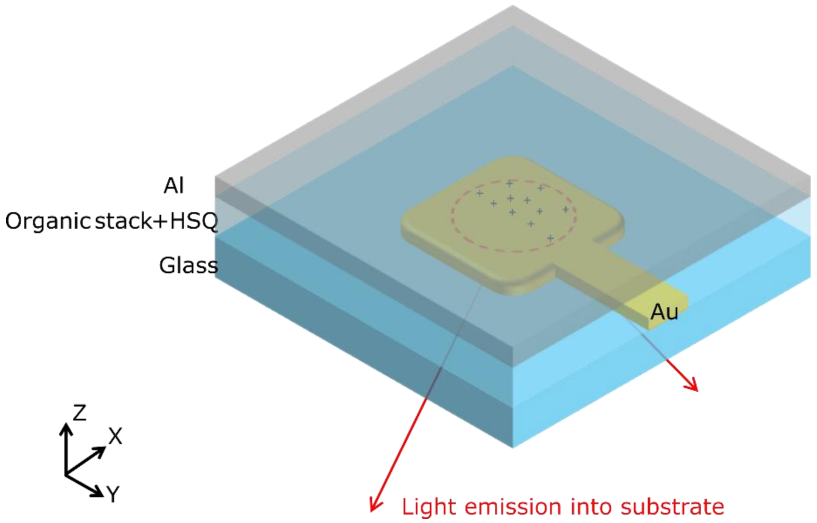

Fig. S11. Configuration of the connected Au nanopatch antenna (300 x 300 nm^2) considered for the light emission simulation model. The Au nanopatch (dielectric according to (*64*)) is 50 nm thick. The connector width is 120 nm. The Al plane (thickness 120 nm, dielectric function according to (*65*)) is located 140 nm above the Au nanopatch. The crosses represent varied electric dipole positions within the nanoaperture, considering structural symmetry, so variations occur within a half-circle region. For the sake of simplicity, the refractive index of the organic stack and the HSQ planarization layer are set to 1.5. The molecular emitters are modeled as electric dipole sources located 50 nm above the Au patch antenna within the emissive layer. We compute the Poynting vector into the glass half-space and integrate it over a solid angle corresponding to the collection cone of the oil-immersion objective (NA = 1.45, polar: 0° to 72°, azimuthal: 0° to 360°). The outcoupling efficiency is then defined as: $\eta_{out}(\omega) = P_{det}(\omega)/P_{tot}(\omega)$, where ω is the optical frequency, and $P_{tot} = P_{det}(\omega) + P_{loss}(\omega)$ is the total power generated by the dipole source. P_{loss} summarizes lossy or undetected channels, for instance, the power directly absorbed by the metal electrodes, trapped waveguided light within the high-index organic stack and surface plasmon polariton modes at the top electrode. To represent the statistical spatial and orientational distribution of excitonic emitters, we average the emission profiles of three orthogonal dipoles (vertical and horizontal) at N positions r_i within the active area defined by the nanoaperture: $\eta_{out}(\omega) = 1/3N\sum[\eta_x(\omega, r_i) + \eta_y(\omega, r_i) + \eta_z(\omega, r_i)]$, where η_x, η_y and η_z are the outcoupling efficiencies with *x*-, *y*- and *z*-polarized dipole excitation, respectively.

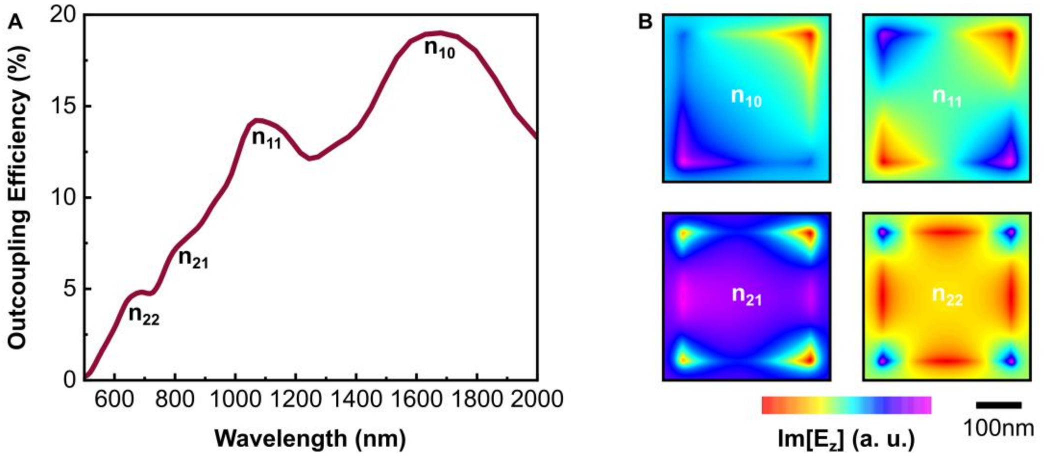

Fig. S12. Plasmonic mode simulation of the Au patch antenna (300 x 300 nm^2) integrated into the device stack. (**A**) Light outcoupling efficiency spectrum with a vertically oriented dipole source positioned laterally off-center by 50 nm along the positive x-axis and thus optimized to excite all the relevant plasmonic modes of the patch antenna. Four distinct resonance peaks are observed at wavelengths of 650 nm, 800 nm, 1100 nm, and 1650 nm, corresponding to the n_{22}, n_{21}, n_{11}, and n_{10} plasmonic modes, respectively (see panel B). The indices refer to the number of nodal planes. The fundamental dipolar n_{10} mode (1650 nm) exhibits the highest radiative efficiency (20 %), with progressively lower efficiencies for higher-order modes (n_{11}: 1100 nm, n_{21}: 800 nm, n_{22}: 650 nm) due to increased field confinement. (**B**) Corresponding plasmonic mode field distribution of the patch antenna: mode n_{10} (1650nm): mode n_{11} (1100nm); mode n_{21} (800nm), and mode n_{22}(650nm). To enhance visualization of the modal field profiles, the fields (Im(E_z)) are depicted for the Au patch antenna in separate simulations that omit the electrical connector.

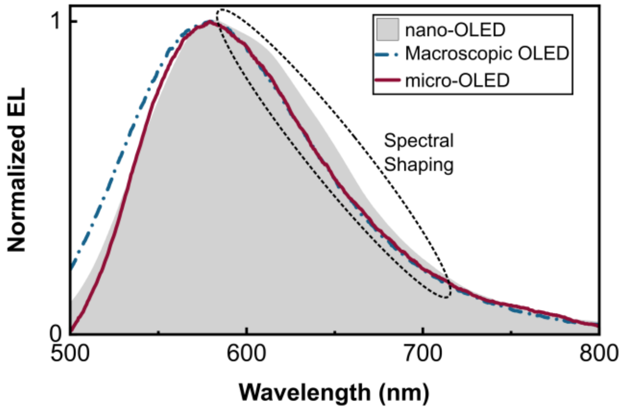

Fig. S13. EL spectra of a nano-OLED, micro-OLED and macroscopic OLED. The nano-OLED is based on 300 x 300 nm^2 Au patch antenna electrodes, the macroscopic OLED (pixel area: 3 mm^2) uses transparent ITO anodes, and the micro-OLED employs 2 x 2 μm^2 Au non-resonant electrodes with an opened HSQ microaperture (diameter of 1.4 µm). Deviations between the nano-/micro-OLED spectra and the macroscopic OLED spectrum below 600 nm are due to the onset of interband absorption in the Au electrodes. In contrast, above 600 nm the distinct spectral shaping by the 300 x 300 nm^2 Au patch antenna modes becomes apparent, while the micro-OLED spectrum closely resembles the macroscopic OLED due to the absence of plasmonic antenna effects.

SI 10: Fabrication Workflow of Hole-Only Nanojunctions

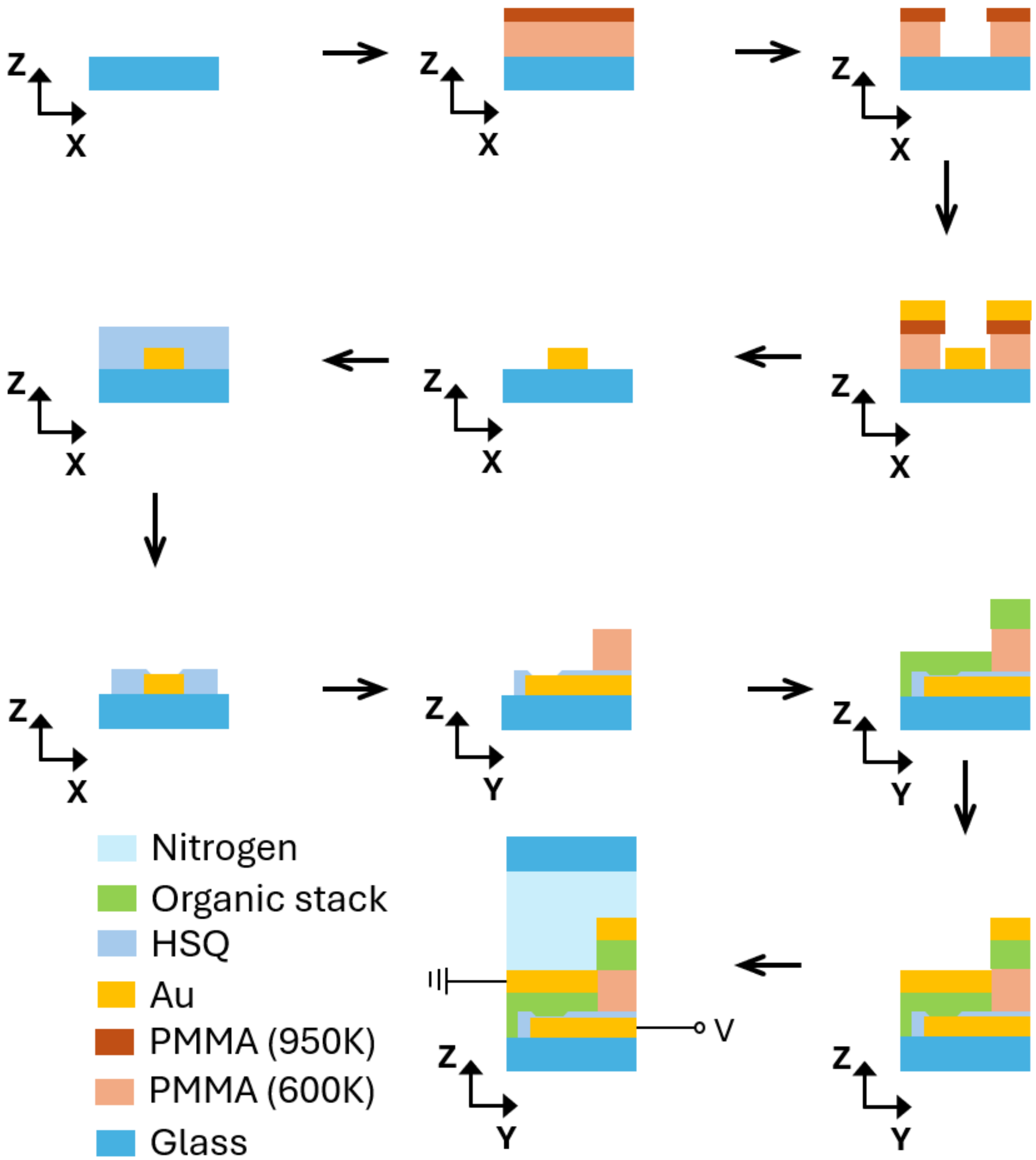

Fig. S14. Fabrication workflow for a hole-only nanojunction. 1. Electrode layout fabrication; 2. Double PMMA layer spincoating; 3. 1st electron-beam writing to pattern the antenna; 4. Thermal evaporation of Au; 5. Lift-off process; 6. HSQ spincoating; 7. Nanoaperture fabrication via the 2nd electron-beam writing; 8. 3rd electron-beam writing to define the pixel with a PMMA insulating layer; 9. Organic stack deposition via stencil lithography; 10. Top metal contact deposition via stencil lithography; 11. Device encapsulation with epoxy resin in a glovebox system.

SI 11: Detection Efficiency of the Electroluminescence Setup

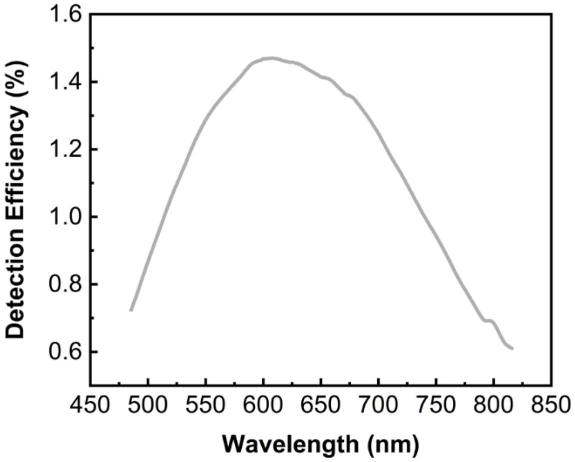

Fig. S15. Detection efficiency of the setup utilized for nano-OLED device characterization including the quantum efficiency of the EMCCD camera, the spectrometer, focusing lens, mirrors and the collection efficiency and transmission of the oil immersion objective.

REFERENCES AND NOTES

1. W. Brütting, S. Berleb, A. G. Mückl, Device physics of organic light-emitting diodes based on molecular materials. *Org. Electron.* **2**, 1–36 (2001).

2. A. M. T. Muthig, O. Mrozek, T. Ferschke, M. Rödel, B. Ewald, J. Kuhnt, C. Lenczyk, J. Pflaum, A. Steffen, Mechano-stimulus and environment-dependent circularly polarized TADF in chiral copper (I) complexes and their application in OLEDs. *J. Am. Chem. Soc.* **145**, 4438–4449 (2023).

3. S. Reineke, M. Thomschke, B. Lüssem, K. Leo, White organic light-emitting diodes: Status and perspective. *Rev. Mod. Phys.* **85**, 1245–1293 (2013).

4. D. Yang, D. Ma, Development of organic semiconductor photodetectors: From mechanism to applications. *Adv. Opt. Mater.* **7**, 1800522 (2019).

5. H. Kleemann, K. Krechan, A. Fischer, K. Leo, A review of vertical organic transistors. *Adv. Funct. Mater.* **30**, 1907113 (2020).

6. F. Mariani, I. Gualandi, W. Schuhmann, E. Scavetta, Micro-and nano-devices for electrochemical sensing. *Microchim. Acta* **189**, 459 (2022).

7. U. Zschieschang, U. Waizmann, J. Weis, J. W. Borchert, H. Klauk, Nanoscale flexible organic thin-film transistors. *Sci. Adv.* **8**, eabm9845 (2022).

8. C. Eckel, J. Lenz, A. Melianas, A. Salleo, R. T. Weitz, Nanoscopic electrolyte-gated vertical organic transistors with low operation voltage and five orders of magnitude switching range for neuromorphic systems. *Nano Lett.* **22**, 973–978 (2022).

9. A. J. Taal, I. Uguz, S. Hillebrandt, C.-K. Moon, V. Andino-Pavlovsky, J. Choi, C. Keum, K. Deisseroth, M. C. Gather, K. L. Shepard, Optogenetic stimulation probes with single-neuron resolution based on organic LEDs monolithically integrated on CMOS. *Nat. Electron.* **6**, 669–679 (2023).

10. W.-J. Joo, J. Kyoung, M. Esfandyarpour, S.-H. Lee, H. Koo, S. Song, Y.-N. Kwon, S. H. Song, J. C. Bae, A. Jo, M.-J. Kwon, S. H. Han, S.-H. Kim, S. Hwang, M. L. Brongersma, Metasurface-driven OLED displays beyond 10,000 pixels per inch. *Science* **370**, 459–463 (2020).

11. S. Hillebrandt, C. K. Moon, A. J. Taal, H. Overhauser, K. L. Shepard, M. C. Gather, High-density integration of ultrabright OLEDs on a miniaturized needle-shaped CMOS backplane. *Adv. Mater.* **36**, e2300578 (2024).

12. C.-M. Kang, J.-W. Shin, S. Choi, B.-H. Kwon, H. Cho, N. S. Cho, J.-I. Lee, S. Kwon, H. Lee, S.-G. Park, in *SID Symposium Digest of Technical Papers* (Wiley Online Library, 2021), vol. 52, pp. 379–382.

13. U. Vogel, B. Beyer, M. Schober, P. Wartenberg, S. Brenner, G. Bunk, S. Ulbricht, P. König, B. Richter, in *SID Symposium Digest of Technical Papers* (Wiley Online Library, 2017), vol. 48, pp. 1125–1128.

14. K. Kato, H. Kobayashi, H. Shishido, T. Isa, T. Aoyama, Y. Jimbo, R. Hodo, K. Kusunoki, H. Kunitake, S. Yamazaki, 5,291-ppi OLED display enabled by monolithic integration of C-axis-aligned crystalline IGZO FET and Si CMOS. *J. Soc. Inf. Disp.* **30**, 690–698 (2022).

15. W. C. Miao, F. H. Hsiao, Y. Sheng, T. Y. Lee, Y. H. Hong, C. W. Tsai, H. L. Chen, Z. Liu, C. L. Lin, R. J. Chung, Microdisplays: Mini-LED, micro-OLED, and micro-LED. *Adv. Opt. Mater.* **12**, 2300112 (2024).

16. S. K. J. Ha, D. Song, S. Park, J. Park, J. Choi, C. Lee, *Information Display* (Wiley, 2023).

17. K. Behrman, I. Kymissis, Micro light-emitting diodes. *Nat. Electron.* **5**, 564–573 (2022).

18. J. M. Smith, R. Ley, M. S. Wong, Y. H. Baek, J. H. Kang, C. H. Kim, M. J. Gordon, S. Nakamura, J. S. Speck, S. P. DenBaars, Comparison of size-dependent characteristics of blue and green InGaN microLEDs down to 1 μm in diameter. *Appl. Phys. Lett.* **116**, (2020).

19. B. Romeira, A. Fiore, Physical limits of nanoleds and nanolasers for optical communications. *Proc. IEEE* **108**, 735–748 (2020).

20. H. S. Wasisto, J. D. Prades, J. Gülink, A. Waag, Beyond solid-state lighting: Miniaturization, hybrid integration, and applications of GaN nano- and micro-LEDs. *Appl. Phys. Rev.* **6**, 041315 (2019).

21. H. Wu, X. Lin, Q. Shuai, Y. Zhu, Y. Fu, X. Liao, Y. Wang, Y. Wang, C. Cheng, Y. Liu, Ultra-high brightness micro-LEDs with wafer-scale uniform GaN-on-silicon epilayers. *Light Sci. Appl.* **13**, 284 (2024).

22. W. J. Baek, J. Park, J. Shim, B. H. Kim, S. Park, H. S. Kim, D.-M. Geum, S. H. Kim, Ultra-low-current driven InGaN blue micro light-emitting diodes for electrically efficient and self-heating relaxed microdisplay. *Nat. Commun.* **14**, 1386 (2023).

23. M. Sheen, Y. Ko, D.-u. Kim, J. Kim, J.-h. Byun, Y. Choi, J. Ha, K. Y. Yeon, D. Kim, J. Jung, Highly efficient blue InGaN nanoscale light-emitting diodes. *Nature* **608**, 56–61 (2022).

24. A. Köhler, H. Bässler, *Electronic Processes in Organic Semiconductors: An Introduction* (John Wiley & Sons, 2015).

25. J. Chen, W. Cranton, M. Fihn, *Handbook of Visual Display Technology* (Springer, 2016).

26. A. Ghosh, B. Corves, *Introduction to Micromechanisms and Microactuators* (Springer, 2015).

27. M. Granström, M. Berggren, O. Inganäs, Micrometer-and nanometer-sized polymeric light-emitting diodes. *Science* **267**, 1479–1481 (1995).

28. F. A. Boroumand, P. W. Fry, D. G. Lidzey, Nanoscale conjugated-polymer light-emitting diodes. *Nano Lett.* **5**, 67–71 (2005).

29. H. Yamamoto, J. Wilkinson, J. P. Long, K. Bussman, J. A. Christodoulides, Z. H. Kafafi, Nanoscale organic light-emitting diodes. *Nano Lett.* **5**, 2485–2488 (2005).

30. J. G. E. Wilbers, B. Xu, P. A. Bobbert, M. P. de Jong, W. G. van der Wiel, Charge transport in nanoscale vertical organic semiconductor pillar devices. *Sci. Rep.* **7**, 41171 (2017).

31. T. Marcato, J. Oh, Z.-H. Lin, S. B. Shivarudraiah, S. Kumar, S. Zeng, C.-J. Shih, Nanomolecular OLED pixelization enabling electroluminescent metasurfaces. arXiv:2404.05336 [physics.optics] (2024).

32. G. D. J. Smit, S. Rogge, T. M. Klapwijk, Enhanced tunneling across nanometer-scale metal–semiconductor interfaces. *Appl. Phys. Lett.* **80**, 2568–2570 (2002).

33. G. Smit, S. Rogge, T. Klapwijk, Scaling of nano-Schottky-diodes. *Appl. Phys. Lett.* **81**, 3852–3854 (2002).

34. M. Rezeq, K. Eledlebi, M. Ismail, R. K. Dey, B. Cui, Theoretical and experimental investigations of nano-Schottky contacts. *J. Appl. Phys.* **120**, 044302 (2016).

35. Y. Shen, N. C. Giebink, Monte Carlo simulations of nanoscale electrical inhomogeneity in organic light-emitting diodes and its impact on their efficiency and lifetime. *Phys. Rev. Appl.* **4**, 054017 (2015).

36. W.-J. Joo, T.-L. Choi, J. Lee, S. K. Lee, M.-S. Jung, N. Kim, J. M. Kim, Metal filament growth in electrically conductive polymers for nonvolatile memory application. *J. Phys. Chem. B.* **110**, 23812–23816 (2006).

37. S. Gao, C. Song, C. Chen, F. Zeng, F. Pan, Dynamic processes of resistive switching in metallic filament-based organic memory devices. *J. Phys. Chem. C* **116**, 17955–17959 (2012).

38. Z. Wang, F. Zeng, J. Yang, C. Chen, F. Pan, Resistive switching induced by metallic filaments formation through poly(3,4-ethylene-dioxythiophene):poly(styrenesulfonate). *ACS Appl. Mater. Interfaces* **4**, 447–453 (2012).

39. A. Hörl, G. Haberfehlner, A. Trügler, F.-P. Schmidt, U. Hohenester, G. Kothleitner, Tomographic imaging of the photonic environment of plasmonic nanoparticles. *Nat. Commun.* **8**, 37 (2017).

40. L. Novotny, B. Hecht, *Principles of Nano-Optics* (Cambridge Univ. Press, 2012).

41. P. Grimm, S. Zeißner, M. Rödel, S. Wiegand, S. Hammer, M. Emmerling, E. Schatz, R. Kullock, J. Pflaum, B. Hecht, Color-switchable subwavelength organic light-emitting antennas. *Nano Lett.* **22**, 1032–1038 (2022).

42. M. Ochs, L. Jucker, M. Rödel, M. Emmerling, R. Kullock, J. Pflaum, M. Mayor, B. Hecht, Site-selective functionalization of in-plane nanoelectrode-antennas. *Nanoscale* **15**, 5249–5256 (2023).

43. M. A. Fusella, R. Saramak, R. Bushati, V. M. Menon, M. S. Weaver, N. J. Thompson, J. J. Brown, Plasmonic enhancement of stability and brightness in organic light-emitting devices. *Nature* **585**, 379–382 (2020).

44. B. Munkhbat, H. Pöhl, P. Denk, T. A. Klar, M. C. Scharber, C. Hrelescu, Performance boost of organic light-emitting diodes with plasmonic nanostars. *Adv. Opt. Mater.* **4**, 772–781 (2016).

45. Y. Qu, M. Slootsky, S. R. Forrest, Enhanced light extraction from organic light-emitting devices using a sub-anode grid. *Nat. Photonics* **9**, 758–763 (2015).

46. P. Pertsch, R. Kullock, V. Gabriel, L. Zurak, M. Emmerling, B. Hecht, Tunable nanoplasmonic photodetectors. *Nano Lett.* **22**, 6982–6987 (2022).

47. R. Kullock, M. Ochs, P. Grimm, M. Emmerling, B. Hecht, Electrically-driven Yagi-Uda antennas for light. *Nat. Commun.* **11**, 115 (2020).

48. M. Ochs, L. Zurak, E. Krauss, J. Meier, M. Emmerling, R. Kullock, B. Hecht, Nanoscale electrical excitation of distinct modes in plasmonic waveguides. *Nano Lett.* **21**, 4225–4230 (2021).

49. M. J. Word, I. Adesida, P. R. Berger, Nanometer-period gratings in hydrogen silsesquioxane fabricated by electron beam lithography. *J. Vac. Sci. Technol. B* **21**, L12–L15 (2003).

50. T. Ferschke, A. Hofmann, W. Brütting, J. Pflaum, Application of fluorescent molecules as noninvasive sensors for optoelectronic characterization on nanometer length scales. *ACS Appl. Electron. Mater.* **2**, 186–194 (2020).

51. A. Kahn, Fermi level, work function and vacuum level. *Mater. Horiz.* **3**, 7–10 (2016).

52. E. Oh, S. Park, J. Jeong, S. J. Kang, H. Lee, Y. Yi, Energy level alignment at the interface of NPB/HAT-CN/graphene for flexible organic light-emitting diodes. *Chem. Phys. Lett.* **668**, 64–68 (2017).

53. Y.-K. Kim, J. Won Kim, Y. Park, Energy level alignment at a charge generation interface between 4, 4′-bis (*N*-phenyl-1-naphthylamino)biphenyl and 1,4,5,8,9,11-hexaazatriphenylene-hexacarbonitrile. *Appl. Phys. Lett.* **94**, 063305 (2009).

54. A. Kahn, N. Koch, W. Gao, Electronic structure and electrical properties of interfaces between metals and π-conjugated molecular films. *J. Polym. Sci. B* **41**, 2529–2548 (2003).

55. N. Turetta, F. Sedona, A. Liscio, M. Sambi, P. Samorì, Au (111) surface contamination in ambient conditions: Unravelling the dynamics of the work function in air. *Adv. Mater. Interfaces* **8**, 2100068 (2021).

56. J. J. M. van der Holst, M. A. Uijttewaal, B. Ramachandhran, R. Coehoorn, P. A. Bobbert, G. A. de Wijs, R. A. de Groot, Modeling and analysis of the three-dimensional current density in sandwich-type single-carrier devices of disordered organic semiconductors. *Phys. Rev. B* **79**, 085203 (2009).

57. A. Fleissner, H. Schmid, C. Melzer, H. von Seggern, Trap-controlled hole transport in small molecule organic semiconductors. *Appl. Phys. Lett.* **91**, 242103 (2007).

58. H. Wang, L. Xie, Q. Peng, L. Meng, Y. Wang, Y. Yi, P. Wang, Novel thermally activated delayed fluorescence materials–Thioxanthone derivatives and their applications for highly efficient OLEDs. *Adv. Mater.* **26**, 5198–5204 (2014).

59. P. Biagioni, J.-S. Huang, B. Hecht, Nanoantennas for visible and infrared radiation. *Rep. Prog. Phys.* **75**, 024402 (2012).

60. Y. Chen, Nanofabrication by electron beam lithography and its applications: A review. *Microelectron. Eng.* **135**, 57–72 (2015).

61. H. Kang, Y. Hwang, C.-m. Kang, J. Y. Kim, C. W. Joo, J.-W. Shin, S. Sim, H. Cho, D. H. Ahn, N. S. Cho, H. M. Youn, Y. J. An, J. S. Kim, C.-W. Byun, H. Lee, Investigating the electrical crosstalk effect between pixels in high-resolution organic light-emitting diode microdisplays. *Sci. Rep.* **13**, 14070 (2023).

62. D. Nečas, P. Klapetek, Gwyddion: An open-source software for SPM data analysis. *Open Phys.* **10**, 181–188 (2012).

63. C.-C. Yang, W.-C. Chen, The structures and properties of hydrogen silsesquioxane (HSQ) films produced by thermal curing. *J. Mater. Chem.* **12**, 1138–1141 (2002).

64. P. B. Johnson, R.-W. Christy, Optical constants of the noble metals. *Phys. Rev. B* **6**, 4370–4379 (1972).

65. E. D. Palik, *Handbook of Optical Constants of Solids* (Academic Press, 1998), vol. 3.
